# Evaluation of energy balances and greenhouse gas emissions from different agricultural production systems in Minqin Oasis, China

**DOI:** 10.7717/peerj.6890

**Published:** 2019-06-26

**Authors:** Zhengang Yan, Wei Li, Tianhai Yan, Shenghua Chang, Fujiang Hou

**Affiliations:** 1 State Key Laboratory of Grassland Agro-Ecosystems, Key Laboratory of Grassland Livestock Industry Innovation, Ministry of Agriculture, China, College of Pastoral Agriculture Science and Technology, Lanzhou University, Lanzhou, Gansu Province, China; 2 College of Information & Science Technology, Gansu Agricultural University, Lanzhou, Gansu Province, China; 3 College of Finance and Economics, Gansu Agricultural University, Lanzhou, Gansu Province, China; 4 Agri-Food and Biosciences Institute, Hillsborough, UK

**Keywords:** Minqin Oasis, Energy balances, Greenhouse gas emissions, Life cycle assessment

## Abstract

Agricultural production in Minqin Oasis, China, is commonly categorized as intensive crop production (ICP), integrated crop–livestock production (ICLP), intensive livestock production (confined feeding) (IFLP), and extensive livestock production (grazing) (EGLP). The objectives of the present study were to use a life cycle assessment technique to evaluate on-farm energy balances and greenhouse gas (GHG) emissions of agricultural production, and to compare the differences among the four systems. Data used in the present study were collected from published literature and face-to face questionnaires from 529 farms in eight towns (two towns per production system) within Minqin county. The ANOVA of averaged data from 2014 to 2015 indicated that the net energy ratio (Output/Input) for the EGLP system was significantly higher than that for any other system (*P* < 0.01), whereas the difference among other three systems were not significant. The EGLP system generated lower CO_2_-eq emissions per hectare of farmland than other systems (*P* < 0.01). Relating carbon economic efficiency to market values (US$) of agricultural products, indicated that the carbon economic efficiency (US$/kg CO_2_-eq) of the IFLP system was significantly greater than that of other systems (*P* < 0.01). The major GHG emission sources varied across the systems, that is, soil respiration is the dominant source in EGLP, while the main sources in IFLP are enteric methane and manure management; in ICLP major sources are enteric methane, soil respiration and fertilizer; and in ICP are soil respiration and fertilizer. The structural equation modelling analysis showed that livestock category was strongly linked to net income. The direct effects and total effects of water use efficiency, via its positive influence on energy balances and GHG emissions were much stronger than those of other dependent variables. The study provides important benchmark information to help develop sustainable agricultural production systems on energy balances and GHG emissions in northwestern China.

## Introduction

Energy is the driving force of existence and is required for agricultural production systems. Studies on energy and greenhouse gas (GHG) emissions are key for analyzing the structure and function of agricultural production systems (Ren, Lin & Wei, 2009). As agricultural production depends heavily on fuel energy and other energy resources, it has a major impact on GHG emissions. This has led to serious environmental problems such as global warming, which has affected the stability and sustainability of agricultural ecosystem, consequently threatening global food security and ecological security (Khoshnevisan et al., 2014). The net energy ratio (NER) is one of the key indicators for developing more sustainable agricultural practices (Ghorbani et al., 2011). High NER in conjunction with low energy use will conserve natural resources, reduce environmental damage, and promote the sustainable development of agriculture. Increasing energy use efficiency are vital for ensuring food and ecological security (Yuan et al., 2018). The NER has been widely used to accurately evaluate energy use and energy use efficiency in various production systems to identify or develop more energy-efficient crop management practices or cropping system at regional, national, and global scales (Yuan et al., 2018).

Agriculture is considered one of the most important global emitters of GHG (Cheng et al., 2011). With the population growth and the large food demand in China, the challenge of reducing GHG emissions is huge. The main sources of GHG emissions are the use of fertilizer and fossil fuel in crop production, and enteric methane and manure management in livestock production. The GHG emissions in China accounted for a large proportion of global emissions in 2014 (Intergovernmental Panel on Climate Change (IPCC), 2014). Similar to other countries, the agricultural emissions mitigation policy in China faces a range of challenges due to the biophysical complexity and heterogeneity of farming systems, as well as other socioeconomic barriers (Wang et al., 2014). At present, the large population and food demand are the main challenges in China. With the rapid development of society, the change in the food structure, and the increase in the quantity of animal-derived food, GHG emissions will increase in China (Dong et al., 2008).

Generally, there are three categories for studying energy balances and GHG emissions from global agricultural production (Hou et al., 2008), that is, crop production, livestock production only, and the combination of crop and livestock production. There is little information available on energy balances and GHG emissions in agricultural production systems in oases in arid regions of China based on production type. Arid regions cover ∼40% of the Earth’s land surface (Reichmann & Sala, 2015). Drying trends may occur most significantly in semi-arid and arid regions as a result of global warming (Huang et al., 2016). The mountain-oasis-desert coupling ecological system is widely distributed in inland areas of the world (Ren & Wan, 1994). Oasis and desert are the dominant ecological landscapes in arid regions of the world, in which water comes from rivers originating from high mountains.

**Figure 1:**
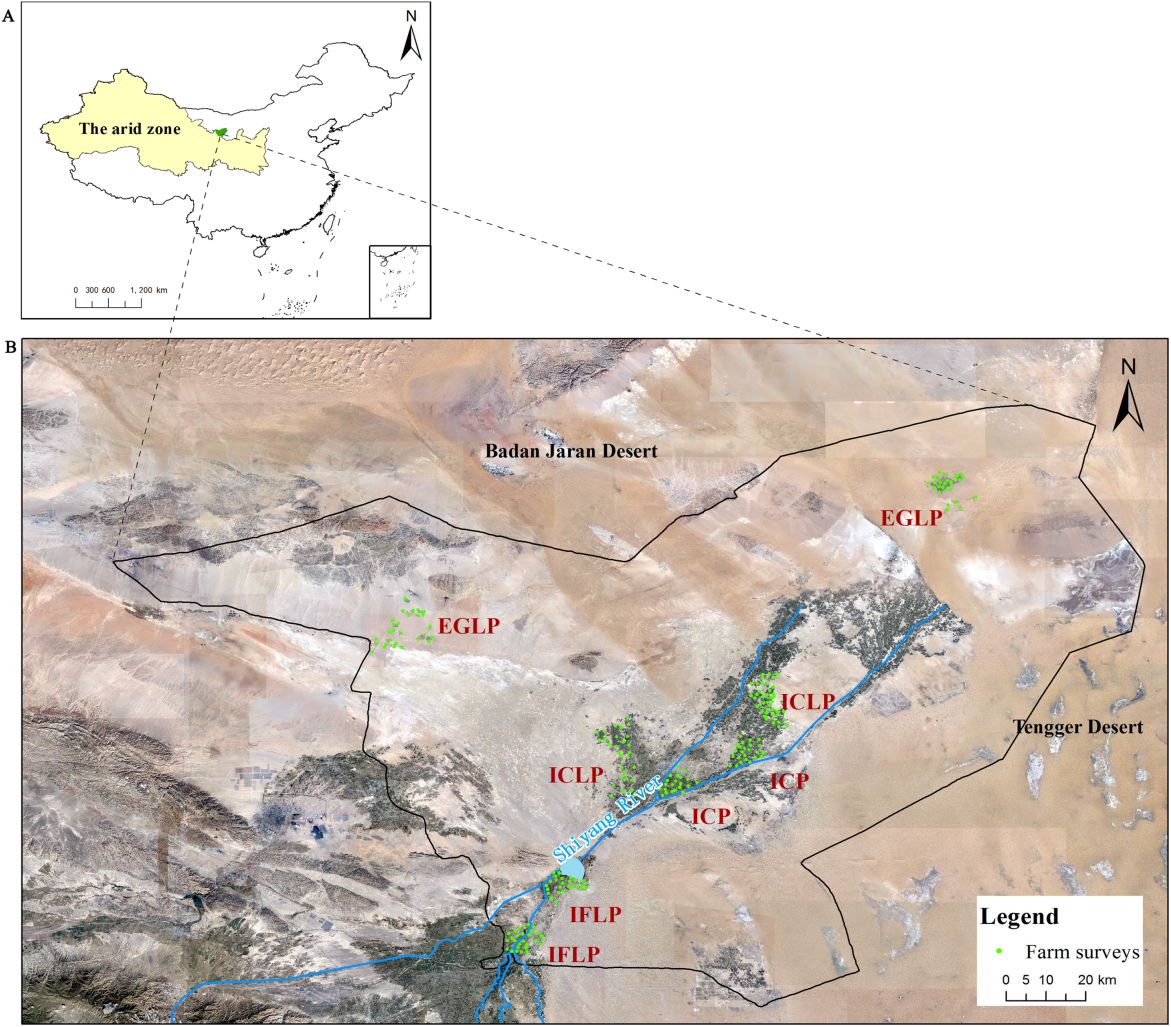
Satellite map of study site at Minqin Oasis, China. (A) Locations of Minqin Oasis; (B) location of study site at Minqin Oasis.

Agricultural production systems in Minqin Oasis surrounded by the Tengger and Badain Jeran Deserts vary greatly in different regions, mainly due to the distribution of water sources located in the Shiyang River, the geography, and other environmental conditions (He et al., 2004). The process and control of desertification in Minqin Oasis are principal modes of action in China and even the world (Hou, Chang & Nan, 2009). Over the past 2,000 years, agricultural production has relied on an extensive grazing system. In history, there are three periods of the opening up of grasslands for planting that resulted in soil desertification in Minqin Oasis. The succession order of agricultural systems in Minqin Oasis is extensive livestock production (grazing) (EGLP), integrated crop–livestock production (ICLP), and intensive crop production (ICP). Agricultural activities of Minqin Oasis, located in northwestern China, are commonly categorized into four contrasting systems: ICP, ICLP, intensive livestock production (confined feeding) (IFLP), and EGLP (Hou, Chang & Nan, 2009). The ICP and IFLP are practiced in well-watered center of Minqin Oasis. The ICLP system is located close to the desert. Grazing in the EGLP system, which is located in the desert, is the main production model (Fig. 1). However, there is no information available on the NER and GHG emissions in Minqin Oasis.

The objectives in this study were to evaluate the difference in energy balances and GHG emissions form 4 contrasting agricultural production systems in Minqin Oasis of China using the life cycle assessment (LCA) technique. These data can offer key information for pursuing low-carbon agriculture and for adjusting the agricultural structure in northwestern China.

## Materials and Methods

The present study was conducted to evaluate the energy balances and GHG emissions within the farm gate using the LCA technique for four contrasting agricultural production systems in Minqin Oasis, China. The LCA technique is recognized as the scientific and appropriate approach to estimate the carbon footprint and quantify the environmental impacts of various aspects of agricultural systems (Hillier et al., 2009; Gollnow et al., 2014; Sanders & Webber, 2014; Pishgar-Komleh, Ghahderijani & Sefeedpari, 2012b). Whereas the potential effects on the environment were mainly caused by mass, and energy flows (Castillo & Mora, 2000). There was high reliability for the evaluated results using the LCA technique compared with other statistical technique, such as input–output model and inventory method (Joint Research Centre of the European Commission (JRC), 2010; Piñero et al., 2018; Intergovernmental Panel on Climate Change (IPCC), 2006).

The LCA technique using a methodological framework to evaluate on farm energy balances and GHG emissions was conducted according to the ISO standard (International Organization for Standardization (ISO). ISO14044, 2006). In this study, the scope and system boundary of LCA only included agricultural production activities on farm. The CH_4_ and N_2_O emission data were converted into CO_2_ equivalents (CO_2_-eq) using their global warming potential (GWP), with GWP of 34 for CH_4_ and 298 for N_2_O for a 100-year period (Intergovernmental Panel on Climate Change (IPCC), 2014). The data used to calculate the GHG emissions were obtained from official records, farm survey data, and published literature.

### Agricultural production systems in Minqin Oasis

**Figure 2:**
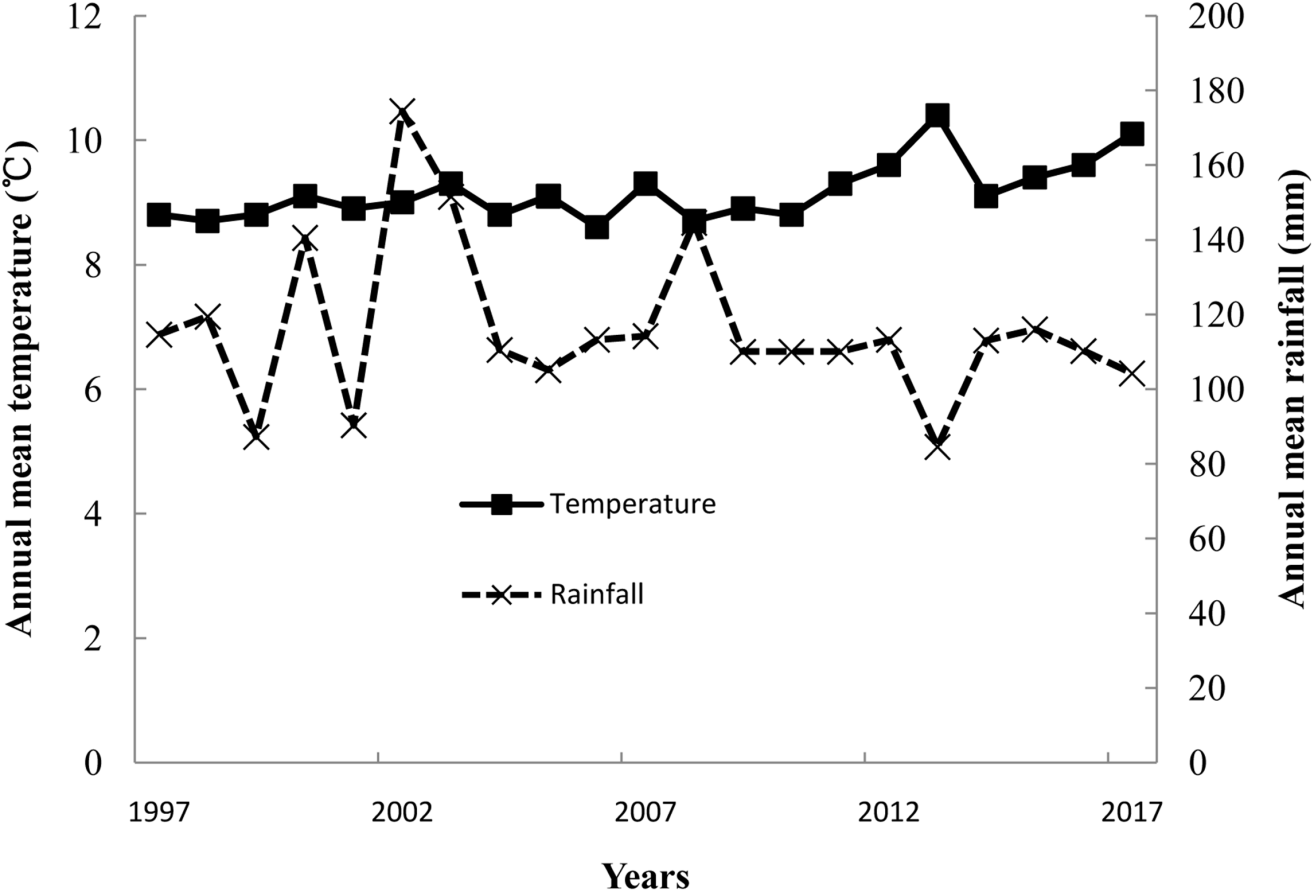
Annual mean temperature and rainfall from 1997 to 2017 in Minqin Oasis.

Minqin Oasis, located in northwestern China (103°05′E, 38°38′N), covers an area of 1.59 × 10^6^ hectares (He et al., 2004). Minqin Oasis has a continental arid climate, and the mean annual temperature, annual frost-free days, and annual rainfall are 7.6 °C, 175 days, and 110.7 mm, respectively. The mean annual rainfall and temperature over the 20-year period from 1997 showed respective decreasing and increasing trends (Fig. 2). Shiyang River, which originates in Qilian Mountain, is the economic lifeblood of Minqin Oasis. The IFLP system has a rich underground water source upstream of Shiyang River for livestock production. However, two of the systems, ICP and ICLP, mainly depend on irrigation, which enables a high input and output of crop production. There was no grazing in the ICLP, and forage fed to livestock was maize, alfalfa hay, and crop straw. Grazing and rangeland are the main production modes at the bottom of the Shiyang River.

To facilitate a comparison of energy balances and GHG emissions from crop and livestock production among the four systems in Minqin Oasis, two typical towns were selected from each production mode to represent the average condition of agricultural production, namely, Caiqi and Chongxing for IFLP; Suwu and Daba for ICLP; Dongba and Shuangzike for ICP; and Hongshagang and Beishan for EGLP (Fig. 1).

### Data collection

Data used in the present study were collected from farm surveys and published literature. The farm surveys were undertaken from 2014 to 2015 with data collected from 529 farmers using a face-to-face questionnaire method in the eight towns selected for the present study (Table 1; Table S1). Over 80% of farmers (434 farmers) selected in 2014 were questioned again in 2015. The questionnaire was designed to collect information on crop and livestock production. The information collected for crop production included the following: labor type and input, crop type, sowing area for each crop, seed source and amount of seeds used, type and rate of fertilizers used in different growth periods, type and rate of pesticide used, fuel consumption for production (ploughing, tillage, transportation, harvesting, and packaging), amount of plastic film, farm machine (type, life, and working hours), electricity consumption for irrigation, yield of crop product, and yield of crop straw. There was no grazing in the ICLP system; forage fed to livestock was from maize and alfalfa produced in crop production. The information for livestock production collected through the farm survey included the following: categories, livestock numbers, age, weight, yields of carcass weight, milk, wool, feed sources, feed usage, lighting of housing structures, and heating of housing structures in winter for livestock management. The mean annual rainfall and temperature during 1997–2017 were derived from agricultural meteorological station in Minqin county. The price of farm products from 2014 to 2015 was obtained from a market survey in each of the study town (Table 2). The local government officials and statisticians told us the price of same farm products between eight towns were consistent in Minqin Oasis in the same year. Structural equation modelling (SEM) was used to estimate the contributions of OtoD (the distance from the oasis to the desert) and OtoM (the distance from the oasis to the mountain) to responses of the soil particle diameter, crop type, livestock category, water use efficiency, net income, energy balances, and GHG emissions. SEM was widely used to evaluate complex causality between variables by translating the hypothesized causal relationships into a pattern of expected statistical relationships in the data (Grace, 2006). The model has a good fit when 0 ≤ χ^2^/d*f* ≤ 2 and 0.05 < *P* ≤ 1. SEM analyses were performed using AMOS 19 (Arbuckle, 2010). The data for SEM were collected from different ways (e.g., soil particle diameter and water use efficiency were collected from public literature; OtoD, OtoM, crop type, livestock category, and net income were collected from farmer interview; carbon balances and GHG emissions were calculated in this study (Table S2).

**Table 1:** Crop and livestock data used in the present study.

	ICP	ICLP	IFLP	EGLP
No. of farm surveys	164	176	126	63
No. of people/household	4–6	4–6	4–6	4–6
Crops (ha/farm)				
Wheat (spring)	0.067–0.133	0.067–0.100	–	–
Maize	0.100–0.133	0.133–0.200	–	–
Cotton	0.133–0.200	0.133–0.200	–	–
Sunflower	0.133–0.200	0.133–2.500	–	–
Alfalfa	0.050–0.067	0.067–0.167	–	–
Chili	0.000–0.033	–	–	–
Tomato	0.000–0.067	0.000–0.067	–	–
Melon	0.033–0.067	0.000–0.033	–	–
Rangeland (ha/farm)	–	–	–	1,350–1,900
Livestock (sheep unit^1^/farm)				
Sheep	–	20–40	785–880	330–349
Dairy cattle	–	–	200–250	–
Beef cattle	–	–	230–275	–

**Note:**

1 Sheep unit (SU) is calculated based on the activity of sheep, one sheep = one SU, one beef cattle = four SU and one dairy cattle = 4.5 SU.

**Table 2:** Average market price of inputs and outputs for agricultural production during the period of 2014 and 2015.

Inputs	CN¥	US$^1^	Outputs	CN¥	US$^1^
Seeds	¥/kg	$/kg	Crop products	¥/kg	$/kg
Wheat (spring)	2.8	0.45	Wheat (spring)	0.75	0.12
Maize	16.00	2.56	Maize	1.90	0.30
Cotton	6.80	1.09	Cotton	6.00	0.96
Sunflower seed	48.00	7.68	Sunflower seed	5.60	0.90
Chili	8.00	1.28	Chili	1.30	0.21
Tomato	20.00	3.20	Tomato	3.00	0.48
Melon	16.00	2.56	Melon	10.00	1.60
Alfalfa	40.00	6.40	Wheat straw	0.70	0.11
Fertilizers			Corn straw	1.96	0.31
Urea	2.00	0.32	Alfalfa straw	1.50	0.24
Mono ammonium phosphate	2.60	0.42	Livestock products		
Phosphate fertilizers	0.50	0.08	Lamb	38.00	6.08
Compound fertilizers	1.60	0.26	Beef	60.00	9.60
Potassium	2.00	0.32	Milk	4.00	0.64.00
Manure	1.00	0.16	Wool	650.00	104.00
Pesticides (¥/kg)					
Herbicides	28.00	4.48			
Insecticides	22.00	3.52			
Fungicides	25.00	4.00			
Mulch					
Plastic mulch	0.77	0.12			
Fuel					
Diesel	12.86	2.06			
Electricity	¥/kwh	$/kwh			
Electricity for irrigation	0.80	0.13			
Feedstuffs	¥/kg	$/kg			
Wheat straw	0.70	0.11			
Corn straw	1.96	0.31			
Alfalfa straw	1.50	0.24			
Corn	1.96	0.31			
Soybean	4.53	0.72			
Wheat husk	1.67	0.27			

**Note:**

1 An average exchange rate of US dollar ($) against Chinese Yuan (¥) for the period of 2014 and 2015 used in the present study was 1:6.25 (Yahoo! Finance, 2019).

### Calculation of energy and GHG emissions from agricultural production

The factors of energy and GHG emissions used in this study were mostly selected from the local literature published in China in recent year using the similar measurement technologies, and from the similar research for the evaluation of energy and GHG emissions of agricultural production in the world.

#### Energy balances of crop and livestock production

For agricultural production systems, the total energy inputs consumed are the human-applied energies classified as direct energy and indirect energy. The energy inputs of the crop production system were estimated using the following Eq. (1).

(1) $$\matrix{ {{\rm{E}}{{\rm{I}}_{{\rm{crop}}}} = \mathop \sum \limits_{i = 1}^n ({\rm{A}}{{\rm{I}}_{{\rm{l}},i}} \times {\rm{E}}{{\rm{F}}_{{\rm{l}},i}} + {\rm{A}}{{\rm{I}}_{{\rm{s}},i}} \times {\rm{E}}{{\rm{F}}_{{\rm{s}},i}} + {\rm{A}}{{\rm{I}}_{{\rm{f}},i}} \times {\rm{E}}{{\rm{F}}_{{\rm{f}},i}} + {\rm{A}}{{\rm{I}}_{{\rm{p}},i}} \times {\rm{E}}{{\rm{F}}_{{\rm{p}},i}} + {\rm{A}}{{\rm{I}}_{{\rm{ie}},i}} \times {\rm{E}}{{\rm{F}}_{{\rm{ie}},i}}} \hskip-5.pc\cr { + {\rm{A}}{{\rm{I}}_{{\rm{pm}},i}} \times {\rm{E}}{{\rm{F}}_{{\rm{pm}},i}} + {\rm{A}}{{\rm{I}}_{{\rm{dc}},i}} \times {\rm{E}}{{\rm{F}}_{{\rm{dc}},i}} + {\rm{A}}{{\rm{I}}_{{\rm{md}},i}} \times {\rm{E}}{{\rm{F}}_{{\rm{md}},i}})} \cr } $$

where EI_crop_, *i*, and *n* represent the energy inputs (MJ/farm), crop type *i*, and number of crops/farm, respectively. AI represents farm inputs, and EF represents energy factors for the crop type *i*: l ∼ labor h/fm (male and female inputs with separate values (Nautiyal et al., 1998); s ∼ seed kg/fm (energy required for seed cleaning and packaging); f ∼ fertilizer kg/fm; p ∼ pesticides kg/fm; ie ∼ electricity for irrigation kW.h/fm (electricity used for on-farm pumping); pm ∼ plastic mulch kg/fm (input fossil fuel energy required for manufacture, transport, and packaging); dc ∼ diesel fuel kg/fm; md ∼ machinery kg/fm (= manufacture energy + fuel consumption energy + depreciation energy) (Table 3). In the field, and the average lifetime of agricultural machinery is 15 years. In the EGLP system, there was no crops for the energy inputs.

**Table 3:** Factors used for calculation of GHG emissions, energy inputs, and energy outputs.

Item	Sub-item	Factors	References
Emission factors of GHG for agricultural production			
Seeds (kg CO_2_-eq/kg)	Wheat (spring)	0.477	West & Marland (2002)
	Maize	3.85	Shi, Chen & Kong (2011a)
	Cotton	2.383	West & Marland (2002)
	Sunflower	0.47	Iriarte & Villalobos (2013)
	Alfalfa	9.643	West & Marland (2002)
	Tomato	1.63	Blook et al. (2010)
	Chili	2.5	The mean of other crops
	Melon	1.9	The mean of other crops
Fertilizers (kg CO_2_-eq/kg)	*N*	6.38	Lu et al. (2008)
	*P*	0.733	Dubey & Lal (2009)
	*K*	0.55	Dubey & Lal (2009)
	Soil emissions CO_2_ after *N* application	0.633	Intergovernmental Panel on Climate Change (IPCC) (2006)
	Soil emissions N_2_O after *N* application	6.205	Adom et al. (2012)
Pesticides (kg CO_2_-eq/kg)	Herbicides	23.1	Lal (2004)
	Insecticides	18.7	Lal (2004)
	Fungicides	13.933	Lal (2010)
Mulch (kg CO_2_-eq/kg)	Plastic mulch	18.993	Cheng et al. (2011)
Electricity (kg CO_2_-eq/kwh)	Electricity for irrigation	0.917	Shi, Chen & Kong (2011a)
Fuel (kg CO_2_-eq/L)	Diesel	2.629	Cheng et al. (2011)
Coal (kg CO_2_-eq/kg)	Fire coal	2.763	Li et al. (2013)
Machinery manufacture (kg CO_2_-eq/kg)	Steel	2.309	Liu et al. (2016)
Machinery depreciation (kg CO_2_-eq/year)	Tractor 7810	14.07	Dyer & Desjardins (2006)
	Tractor 55/60	0.49	Dyer & Desjardins (2006)
	Tractor 1002/1202	1.32	Dyer & Desjardins (2006)
	Tractor 250	0.16	Dyer & Desjardins (2006)
	Harvester 1200	0.66	Dyer & Desjardins (2006)
	Harvester 154	1.34	Dyer & Desjardins (2006)
Feed processing (kg CO_2_-eq/kg)	Maize	0.0102	Meng et al. (2014)
	Soybean	0.1013	Meng et al. (2014)
	Wheat	0.0319	Meng et al. (2014)
CH_4_ emissions from enteric fermentation (kg CO_2_-eq/head/year)	Sheep	170	Intergovernmental Panel on Climate Change (IPCC) (2014)
	Beef cattle	1,598	Intergovernmental Panel on Climate Change (IPCC) (2014)
	Dairy cattle	2,074	Intergovernmental Panel on Climate Change (IPCC) (2014)
CH_4_ emissions from manure management (kg CO_2_-eq/head/year)	Sheep	3.74	Intergovernmental Panel on Climate Change (IPCC) (2014)
	Beef cattle	34	Intergovernmental Panel on Climate Change (IPCC) (2014)
	Dairy cattle	340	Intergovernmental Panel on Climate Change (IPCC) (2014)
N_2_O emissions from manure management (kg CO_2_-eq/head/year)	Sheep	62.3	Intergovernmental Panel on Climate Change (IPCC) (2014)
	Beef cattle	120.4	Intergovernmental Panel on Climate Change (IPCC) (2014)
	Dairy cattle	106.7	Intergovernmental Panel on Climate Change (IPCC) (2014)
Energy factors of agricultural production inputs			
Seeds (MJ/kg)	Wheat (spring)	17.9	Dazhong & Pimentel (1984)
	Maize	104.65	Pimentel (1980)
	Cotton	22.024	Huang, Yang & Li (2004)
	Sunflower	38.312	The mean of other crops
	Alfalfa	108.82	Dazhong & Pimentel (1984)
	Tomato	16.33	Lu (1994)
	Chili	1.5	Ozkan, Akcaoz & Fert (2004)
	Melon	2.3	Ozkan, Akcaoz & Fert (2004)
Fertilizers (MJ/kg)	*N*	78.1	Pimentel (1980)
	*P*	17.4	Pimentel (1980)
	*K*	13.7	Pimentel (1980)
Farmyard manure (MJ/kg)	Animal manure	14.63	Dazhong & Pimentel (1984)
Pesticides (MJ/kg)	Herbicides	278	Pimentel (1980)
	Insecticides	233	Pimentel (1980)
	Fungicides	121	Pimentel (1980)
Mulch (MJ/kg)	Plastic mulch	51.9	Cheng et al. (2011)
Fuel (MJ/kg)	Diesel	47.78	Cheng et al. (2011)
Electricity (MJ/kwh)	Electricity for irrigation and lighting	12	Pimentel (1980)
Machinery manufacture (MJ/kg)	Agricultural machinery	86.77	Pimentel (1980)
Machinery depreciation (MJ/kg/year)	Agricultural machinery	5.21	Dazhong & Pimentel (1984)
Coal (MJ/kg)	Fire coal	22.28	Liu et al. (2017)
Human labor (MJ/h)	Male	0.68	Nautiyal et al. (1998)
	Female	0.52	Nautiyal et al. (1998)
Forage feed (MJ/kg)	Wheat hay	15.05	Wang et al. (2004)
	Maizehay	15.22	Wang et al. (2004)
	Alfalfa hay	18.8	Wang et al. (2004)
Concentrate feed (MJ/kg)	Maize	18.26	Wang et al. (2004)
	Soybean	18.83	Wang et al. (2004)
	Wheat husk	13.72	Wang et al. (2004)
Energy factors of agricultural products			
Grain (MJ/kg)	Wheat (spring)	12.56	Wang et al. (2004)
	Maize	18.26	Wang et al. (2004)
	Cotton	22.024	Huang, Yang & Li (2004)
	Sunflower	10.4	The mean of other crops
	Tomato	1.258	Huang, Yang & Li (2004)
	Chili	1.258	Huang, Yang & Li (2004)
	Melon	1.6722	Huang, Yang & Li (2004)
Hay (MJ/kg)	Wheat (spring)	15.05	Wang et al. (2004)
	Maize	15.22	Wang et al. (2004)
	Alfalfa	18.8	Wang et al. (2004)
	Cotton	18.3	Wang et al. (2017)
Livestock products (MJ/kg)	Lamb	12.877	Huang, Yang & Li (2004)
	Beef	13.88	Huang, Yang & Li (2004)
	Milk	2.889	Huang, Yang & Li (2004)
	Wool	23.41	Dazhong & Pimentel (1984)

The energy output of the crop refers to the energy density of that product including the grain, straw, and root. The energy outputs for each type of crop are calculated using Eq. (2).

(2) $${\rm{E}}{{\rm{O}}_{{\rm{crop}}}} = \sum\limits_{i = 1}^n {({Y_{{\rm{grain}},i}}} \times {\rm{E}}{{\rm{F}}_{{\rm{grain}},i}} + {Y_{{\rm{straw}},i}} \times {\rm{E}}{{\rm{F}}_{{\rm{straw}},i}} + {Y_{{\rm{root}},i}} \times {\rm{E}}{{\rm{F}}_{{\rm{root}},i}})$$

where EO_crop_, *i*, and *n* represent the energy outputs (MJ/farm), crop type *i*, and number of crops/farm, respectively. *Y* represents crop yield (kg/farm), and EF represents energy factors for the crop type *i*: grain ∼ crop grain kg/fm; straw ∼ crop straw kg/fm; root ∼ crop root kg/fm (Table 3).

For livestock production, input energies included feed production and processing, labor inputs, electrify and fuel (electricity and coal) inputs for housing structures. The output energies were carcass, milk, and wool. The energy inputs for each category of livestock are calculated using Eq. (3).

(3) $$\eqalign{ & {\rm{E}}{{\rm{I}}_{{\rm{livestock}}}} = \sum\limits_{i = 1}^n {(\sum\limits_{j = 1}^m {({\rm{F}}{{\rm{I}}_{{\rm{feed}},j}}} }  \times {\rm{E}}{{\rm{F}}_{{\rm{feed}},j}}{)_i} + {\rm{L}}{{\rm{I}}_{{\rm{labor}},i}} \times {\rm{E}}{{\rm{F}}_{{\rm{labor}},i}} + {\rm{HM}}{{\rm{I}}_{{\rm{elec}},i}} \times {\rm{E}}{{\rm{F}}_{{\rm{elec}},i}}  \cr & \quad \quad \quad \quad \quad \, + {\rm{HM}}{{\rm{I}}_{{\rm{coal}},i}} \times {\rm{E}}{{\rm{F}}_{{\rm{coal}},i}}) \cr} $$

where EI_livestock_, *i*, *n*, *j*, and *m* represent the energy inputs (MJ/farm), livestock category *i*, number of livestocks/farm, feed type *j*, and number of feeds/farm, respectively. FI_feed,*j*_, and EF_feed,*j*_ represent feed input classified as *j* (kg/farm), and energy factor of the feed classified as *j*, respectively. LI_labor,*i*_, HMI_elec,*i*_, and HMI_coal,*i*_ represent the energy input of livestock classified as *i* for human labor (h/farm), lighting of housing structures (kW.h/farm), and heating of housing structures in winter for livestock management (kg/farm), respectively. EF_labor,*i*_, EF_elec,*i*_, and EF_coal,*i*_ represent the energy factors of livestock classified as *i* for labor, electricity and coal, respectively (Table 3). In the EGLP system, the energy input only included inputs of supplementary feeding in winter.

The energy outputs for each category of livestock are calculated using Eq. (4).

(4) $${\rm{E}}{{\rm{O}}_{{\rm{livestock}}}} = \sum\limits_{i = 1}^n {({Y_{{\rm{carcass}},i}}} \times {\rm{E}}{{\rm{F}}_{{\rm{carcass}},i}} + {Y_{{\rm{milk}},i}} \times {\rm{E}}{{\rm{F}}_{{\rm{milk}},i}} + {Y_{{\rm{wool}},i}} \times {\rm{E}}{{\rm{F}}_{{\rm{wool}},i}})$$

where EO_livestock_, *i*, and *n* represent energy output (MJ/farm), livestock category *i*, and number of livestocks/farm, respectively. *Y* represents the yield of livestock product (kg/farm), and EF represents energy factors for the livestock category *i*: carcass ∼ livestock carcass kg/fm; milk ∼ dairy milk kg/fm; wool ∼ sheep wool kg/fm (Table 3). Based on the energy balances of the inputs and outputs, the energy balances and NER were calculated as follows:

(5) $${\rm{E}}{{\rm{B}}_{{\rm{farm}}}} = ({\rm{E}}{{\rm{O}}_{{\rm{crop}}}} + {\rm{E}}{{\rm{O}}_{{\rm{livestock}}}}) - ({\rm{E}}{{\rm{I}}_{{\rm{crop}}}} + {\rm{E}}{{\rm{I}}_{{\rm{livestock}}}})$$

(6) $${\rm{NE}}{{\rm{R}}_{{\rm{farm}}}} = {{{\rm{E}}{{\rm{O}}_{{\rm{crop}}}} + {\rm{E}}{{\rm{O}}_{{\rm{livestock}}}}} \over {{\rm{E}}{{\rm{I}}_{{\rm{crop}}}} + {\rm{E}}{{\rm{I}}_{{\rm{livestock}}}}}}$$

where EB_farm_, and NER_farm_ represent the respective energy balances (MJ/farm) and the NER (Output/Input) of agricultural production systems in Minqin Oasis. EO_crop_, EO_livestock_, EI_crop_, and EI_livestock_ represent the same parameters as in the previous equations.

#### GHG emissions from crop production and rangeland (not including livestock)

The GHG emissions from crop production and pasture (rangeland) were estimated using the LCA technique in the following Eq. (7).

(7) $$\eqalign{{\rm{C}}{{\rm{E}}_{{\rm{crop\&rangeland}}}} = \sum\limits_{i = 1}^n ( {\rm{A}}{{\rm{I}}_{{\rm{s}},i}} \times {\rm{E}}{{\rm{F}}_{{\rm{s}},i}} + {\rm{A}}{{\rm{I}}_{{\rm{f}},i}} \times {\rm{E}}{{\rm{F}}_{{\rm{f}},i}} + {\rm{A}}{{\rm{I}}_{{\rm{p}},i}} \times {\rm{E}}{{\rm{F}}_{{\rm{p}},i}} + {\rm{A}}{{\rm{I}}_{{\rm{ie}},i}} \times {\rm{E}}{{\rm{F}}_{{\rm{ie}},i}} \hskip 8pt\cr + {\rm{A}}{{\rm{I}}_{{\rm{pm}},i}} \times {\rm{E}}{{\rm{F}}_{{\rm{pm}},i}} + {\rm{A}}{{\rm{I}}_{{\rm{dc}},i}} \times {\rm{E}}{{\rm{F}}_{{\rm{dc}},i}} + {\rm{A}}{{\rm{I}}_{{\rm{md}},i}} \times {\rm{E}}{{\rm{F}}_{{\rm{md}},i}} + {\rm{SOI}}{{\rm{L}}_{{\rm{res}},i}}) \cr} $$

where CE_crop&rangeland_, *i*, and *n* represent GHG emissions from crop production and pasture (kg CO_2_-eq/farm), crop type *i*, and number of crops/farms, respectively. AI represents farm inputs, and EF represents emission factors for the crop type *i*: s ∼ seed kg/fm (GHG emissions from seed cleaning and packaging); f ∼ fertilizer kg/fm; p ∼ pesticides kg/fm; ie ∼ electricity for irrigation kW.h/fm (GHG emissions from electricity used for on-farm pumping); pm ∼ plastic film kg/fm (GHG emissions from manufacture, transport, and packaging); dc ∼ diesel fuel L/fm; md ∼ machinery kg/fm (= GHG emissions from machinery manufacture + fuel consumption + machinery depreciation) (Table 3). In the field, the average lifetime of agricultural machinery is 15 years. The value of the emission factor for the above production input was calculated in the same way as the energy factor. SOIL_res_ only represents GHG emissions from soil respiration using the following Eq. (8) (Chen et al., 2010). For the EGLP system, GHG emissions from soil have been listed under crop and rangeland (Table 4; Table S3), and are calculated for soil respiration only.

**Table 4:** GHG emissions, carbon stocks, carbon balances, and carbon economic efficiency of agricultural production systems in Minqin Oasis.

	ICP	ICLP	IFLP	EGLP	SED^1^	*P*-Value
Carbon balances^2^ (ton CO_2_-eq/farm)						
Crop and Rangeland (not including livestock)						
GHG emissions^3^	1.05^b^	1.01^b^	–	3.40^a^	0.004	<0.001
Carbon stock^4^	0.94^b^	0.97^b^	–	4.59^a^	0.005	<0.001
Carbon balance^5^	−0.11^c^	−0.03^b^	–	0.59^a^	0.002	<0.001
Livestock						
GHG emissions^6^	–	0.92^c^	2.28^a^	1.90^b^	0.006	<0.001
Carbon stock^7^	–	0.10^c^	0.87^a^	0.57^b^	0.003	<0.001
Carbon balance^8^	–	−0.82^a^	−1.41^c^	−1.34^b^	0.005	<0.001
Crop and Rangeland (including livestock)						
GHG emissions	1.05^c^	1.24^c^	2.28^b^	4.00^a^	0.005	<0.001
Carbon stock	0.94^b^	0.98^b^	0.87^b^	4.60^a^	0.005	<0.001
Carbon balance	−0.11^b^	−0.26^b^	−1.41^c^	0.59^a^	0.004	<0.001
Carbon economic efficiency^9^						
CN¥ (1,000¥/ton CO_2_-eq/farm)						
Crop and Rangeland (not including livestock)	0.78^a^	0.79^a^	–	0.26^b^	0.004	<0.001
Livestock	–	3.47^a^	3.39^b^	3.48^a^	0.017	<0.001
Crop and Rangeland (including livestock)	0.78^b^	1.05^b^	3.39^a^	1.97^b^	0.014	<0.001
US$^10^ (160$/ton CO_2_-eq/farm)						
Crop and Rangeland (not including livestock)	0.12^a^	0.13^a^	–	0.04^b^	0.001	<0.001
Livestock	–	0.56^a^	0.54^b^	0.56^a^	0.003	<0.001
Crop and Rangeland (including livestock)	0.12^b^	0.17^b^	0.54^a^	0.32^b^	0.002	<0.001
Crop and Rangeland (not including livestock) (ton CO_2_-eq/ha)						
GHG emissions	12.7^a^	12.6^a^	–	5.6^b^	0.04	<0.001
Carbon stock	9.6^c^	12.1^b^	–	22.2^a^	0.08	<0.001
Carbon balance	−3.2^c^	−0.6^b^	–	16.6^a^	0.07	<0.001

**Notes:**

1 SED, standard error of differences.

2 The data of carbon balance (GHG emissions, Carbon stock) were transformed to ensure homogeneity of variance using equation log_10_ (data+1).

3 GHG emissions from crop production inputs.

4 Carbon stock of the net accumulation of photosynthesis from crop products, such as the grain, stem, and root.

5 Carbon balances of crop production (carbon stock—GHG emissions).

6 GHG emissions from livestock production.

7 Carbon stock from livestock products, such as the carcass, milk and wool.

8 Carbon balances of livestock production (carbon stock—GHG emissions).

9 The data of carbon economic efficiency (CN¥, US$) were transformed to ensure homogeneity of variance using equation log_10_ (data+1).

10 US$: An average of the US$: CN¥ exchange rate for the years 2014–2015 of 1 US$: 6.25 CN¥ has been used to show prices in both currencies (Yahoo! Finance, 2019); similar letters: no significant difference; dissimilar letters (a, b, c) indicates a significant difference (*P* < 0.05).

(8) $${\rm{SOI}}{{\rm{L}}_{{\rm{res}}}} = {R_0} \times {e^{{\rm{QT}}}} \times {{P \times {\rm{SOC}}} \over {(P + K) \times ({\rm{SOC}} + \rm\psi)}}$$

In this equation, each variable denotes the following:

1. SOIL_res_: GHG emissions of heterotrophic respiration from the soil (kg/C/m^2^/year);
2. *R*_0_: the soil respiration at 0 °C without precipitation limitation (kg/C/m^2^/year);
3. *Q*: the exponential relation between soil respiration and temperature (°C^−1^);
4. *T*: the mean annual temperature (°C);
5. *P*: the annual rainfall (m);
6. *K*: the half-saturation constant of the hyperbolic relationship of soil respiration with annual precipitation (m);
7. ψ: the half-saturation constant of the hyperbolic relationship of soil respiration with soil organic carbon storage (kg/C/m^2^);
8. SOC: organic carbon values of soil at a depth between 0 and 20 cm (kg/C/m^2^).

In this study, the parameter value of cropland (*R*_0_ = 4.63, *Q* = 0.004, *T* = 9.25, *P* = 0.115, *K* = 1.94, ψ = 4.27, SOC = 5.09) differ from the corresponding data of grassland (*R*_0_ = 9.62, *Q* = 0.023, *T* = 9.25, *P* = 0.115, *K* = 5.16, ψ = 3.99, SOC = 2.86) (Chen et al., 2010; Chen, Gai & Li, 2009).

The carbon stock of both crop and pasture (rangeland) refers to the carbon stock expressed as CO_2_-eq, which is the net accumulation of photosynthetic products. The carbon stock of both crop and pasture is calculated using Eq. (9) (Shi et al., 2011b).

(9) $${\rm{C}}{{\rm{S}}_{{\rm{crop\& rangeland}}}} = \sum\limits_{i = 1}^n {({\rm{C}}{{\rm{S}}_{{\rm{grain}},i}} + {\rm{C}}{{\rm{S}}_{{\rm{stem}},i}} + {\rm{C}}{{\rm{S}}_{{\rm{root}},i}})} $$

where CS_crop&rangeland_, *i*, *n*, CS_grain,*i*_, CS_stem,*i*_, and CS_root,*i*_ represent the carbon values (kg CO_2_-eq/farm) accumulated in the plant (crop and grass) and soil in the process of plant (crop and grass) production, plant (crop and grass) type *i*, number of plants (crop and grass)/farm, carbon stock of grain (kg CO_2_-eq/farm), stem (kg CO_2_-eq/farm), and root (kg CO_2_-eq/farm) for plant (crop and grass) type *i*, respectively. The values of CS_grain_, CS_stem_, and CS_root_ were calculated using Eqs. (10–12) (Shi et al., 2011b). In order to evaluate the allocation of carbon to plant parts in the grain crop, the carbon concentration of all plants parts was assumed to be 0.45 g/g (Yousefi, Damghani & Khoramivafa, 2014).

(10) $${\rm{C}}{{\rm{S}}_{{\rm{grain}}}} = \sum\limits_{i = 1}^n {{\rm{Yiel}}{{\rm{d}}_i}} \times (1 - {\rm{W}}{{\rm{C}}_i}) \times 0.45$$

(11) $${\rm{C}}{{\rm{S}}_{{\rm{stem}}}} = \sum\limits_{i = 1}^n {\left( {{{{\rm{C}}{{\rm{S}}_{{\rm{grain}},i}}} \over {{H_i} - {\rm{C}}{{\rm{S}}_{{\rm{grain}},i}}}}} \right)} $$

(12) $${\rm{C}}{{\rm{S}}_{{\rm{root}}}} = \sum\limits_{i = 1}^n ({\rm{C}}{{\rm{S}}_{{\rm{grain,}}i}} + {\rm{C}}{{\rm{S}}_{stem,i}}) \times {R_i}$$

where CS_grain_, CS_stem_, CS_root_, Yield_*i*_, WC_*i*_, CS_grain,*i*_, CS_stem,*i*_, *H_i_*, *R_i_*, *i*, and *n* represent the carbon stock of plant (crop and grass) grain (kg CO_2_-eq/farm), stem (kg CO_2_-eq/farm), and root (kg CO_2_-eq/farm), the yield of plant classified as *i* (kg/farm), the water content of the plant classified as *i* (%), the carbon stock of the plant grain (kg CO_2_-eq/farm), stem (kg CO_2_-eq/farm), and root (kg CO_2_-eq/farm) classified as *i*, the harvest index of the plant classified as *i* (%), the root-shoot ratio classified as *i* (%), plant type *i*, and number of plants classified as *i* (Table 5).

**Table 5:** Parameters used in the present study to calculate carbon stocks for crop and pasture (rangeland) production.

Crops	Harvest index (%)	Water content (%)	Root-shoot ratio (%)	References
Wheat (spring)	40	13	14	Tian & Zhang (2013)
Corn	40	14	16	Tian & Zhang (2013)
Cotton	38.3	9	19	Tian & Zhang (2013)
Sunflower	31	10	30.6	Miao et al. (1998)
Tomato	60	90	–	Tian & Zhang (2013)
Chili	60	90	–	Tian & Zhang (2013)
Melon	70	90	–	Tian & Zhang (2013)
Alfalfa	35	83	0.178	Qi et al. (2011)
Grass (rangeland)	35	83	7.7	Ni (2001)

The carbon balances of crop production are calculated using Eq. (13).

(13) $${\rm{C}}{{\rm{B}}_{{\rm{crop\& rangeland}}}} = {\rm{C}}{{\rm{S}}_{{\rm{crop\& rangeland}}}} - {\rm{C}}{{\rm{E}}_{{\rm{crop\& rangeland}}}}$$

where CB_crop&rangeland_, CS_crop&rangeland_, and CE_crop&rangeland_ represent the respective carbon balances (kg CO_2_-eq/farm), carbon stocks and GHG emissions from inputs of crop production and pasture. If the value of CB_crop&rangeland_ is greater than zero, the agricultural production system is a carbon sink.

#### GHG emissions from livestock production

Annual GHG emissions from inputs for each class of livestock were calculated in terms of the following: feed production and processing, lighting electricity, coal inputs, enteric fermentation, and manure management. The GHG emissions from livestock production are calculated using Eq. (14).

(14) $$\displaylines{{\rm{C}}{{\rm{E}}_{{\rm{livestock}}}} = \sum\limits_{i = 1}^n {\bigg(\sum\limits_{j = 1}^m {({\rm{F}}{{\rm{I}}_{{\rm{feed}},j}}} } \times {\rm{E}}{{\rm{F}}_{{\rm{feed}},j}}{)_i} + {\rm{HM}}{{\rm{I}}_{{\rm{elec}},i}} \times {\rm{E}}{{\rm{F}}_{{\rm{elec}},i}} + {\rm{HM}}{{\rm{I}}_{{\rm{coal}},i}} \times {\rm{E}}{{\rm{F}}_{{\rm{coal}},i}} \cr\hskip 3.9pc +\ {\rm{NU}}{{\rm{M}}_{{\rm{livestock}},i}} \times ({\rm{E}}{{\rm{F}}_{{\rm{C}}{{\rm{H}}_{\rm{4}}}}}_{ - {\rm{Enteric}},i} + {\rm{E}}{{\rm{F}}_{{\rm{C}}{{\rm{H}}_{\rm{4}}}}}_{ - {\rm{Manure}},i} + {\rm{E}}{{\rm{F}}_{{{\rm{N}}_{\rm{2}}}{\rm{O}}}}_{\, - {\rm{Manure}},i})\bigg) \cr} $$

where CE_livestock_, *i*, *n*, *j*, and *m* represent the GHG emissions of livestock (kg CO_2_-eq/farm), livestock category *i*, number of livestocks/farm, feed type *j*, and number of feeds/farm, respectively. FI_feed,*j*_, and EF_feed,*j*_ represent feed input classified as *j* (kg/farm), and emission factor of the feed classified as *j*, respectively. HMI_elec,*i*_, HMI_coal,*i*_, and NUM_livestock,*i*_, represent the farm input of livestock classified as *i* for lighting of housing structures (kW.h/farm), heating of housing structures in winter for livestock management (kg/farm), and number of livestock category *i* (head/farm), respectively. EF_elec,*i*_, EF_coal,*i*_, ${\rm{E}}{{\rm{F}}_{{\rm{C}}{{\rm{H}}_{\rm{4}}}}}_{ - {\rm{Enteric}},i}$, ${\rm{E}}{{\rm{F}}_{{\rm{C}}{{\rm{H}}_{\rm{4}}}}}_{ - {\rm{Manure}},i}$, ${\rm{E}}{{\rm{F}}_{{{\rm{N}}_{\rm{2}}}{\rm{O}}}}_{\, - {\rm{Manure}},i}$ represent the emission factors of livestock classified as *i* for electricity, coal, CH_4_ emissions from ruminant enteric fermentation, CH_4_ emissions from manure management, and N_2_O emissions from manure management, respectively (Table 3). The value of CH_4_ and N_2_O emissions from ruminant enteric fermentation and manure management are all expressed as CO_2_-eq (Table 3).

The carbon stock (accumulation) of livestock production mainly included carbon stock expressed as CO_2_-eq from livestock products, such as the carcass, milk, and wool. The carbon stock of livestock is calculated using Eq. (15) (Wu, Gao & Hou, 2017).

(15) $${\rm{C}}{{\rm{S}}_{{\rm{livestock}}}} = \sum\limits_{i = 1}^n {{\rm{C}}{{\rm{S}}_i} = \sum\limits_{i = 1}^n {({\rm{L}}{{\rm{W}}_i} \times 0.2)} } $$

where CS_livestock_, *i*, *n*, CS_*i*_, and LW_*i*_ represent the carbon stock (kg CO_2_-eq/farm), livestock category *i*, livestock numbers classified as *i* (head/farm), carbon stock of livestock classified as *i* (kg CO_2_-eq/farm), and live weight of livestock numbers classified as *i* (kg CO_2_-eq/farm).

The carbon balances of livestock production are calculated using Eq. (16).

(16) $${\rm{C}}{{\rm{B}}_{{\rm{livestock}}}} = {\rm{C}}{{\rm{S}}_{{\rm{livestock}}}} - {\rm{ C}}{{\rm{E}}_{{\rm{livestock}}}}$$

where CB_livestock_, CS_livestock_, and CE_livestock_ represent carbon balances (kg CO_2_-eq/farm), carbon stocks (kg CO_2_-eq/farm) and GHG emissions (kg CO_2_-eq/farm) of livestock production inputs, respectively. If the value of CB_livestock_ is less than zero, the livestock production system is a carbon source.

#### Carbon balances of agricultural production systems

In brief, the carbon balances of agricultural production systems in Minqin Oasis are calculated using Eq. (17).

(17) $${\rm{C}}{{\rm{B}}_{{\rm{farm}}}} = ({\rm{C}}{{\rm{S}}_{{\rm{crop\& rangeland}}}} + {\rm{C}}{{\rm{S}}_{{\rm{livestock}}}}) - ({\rm{C}}{{\rm{E}}_{{\rm{crop\& rangeland}}}} + {\rm{C}}{{\rm{E}}_{{\rm{livestock}}}})$$

where CB_farm_ represents carbon balances (kg CO_2_-eq/farm) of agricultural production systems in Minqin Oasis. CS_crop&rangeland_, CS_livestock_, CE_crop&rangeland_, and CE_livestock_ represent the same parameters as in the above equations. Values of CB_farm_ greater than zero, equal to zero, and less than zero indicate that the agricultural production system is a carbon source, a balanced carbon status or a carbon sink, respectively.

#### Calculation of carbon economic efficiency

The total carbon economic efficiency (¥, Chinese currency) associated with the emissions of one kg of carbon from crop or livestock products was calculated using Eq. (18) (Shi et al., 2011b).

(18) $${\rm{CE}}{{\rm{E}}_{{\rm{farm}}}} = {{\sum\nolimits_{i = 1}^n {({\rm{Y}}{{\rm{P}}_{{\rm{product}}(i)}} \times {\rm{PRIC}}{{\rm{E}}_{{\rm{product}}(i)}})} } \over {{\rm{C}}{{\rm{E}}_{{\rm{crop}}}} + {\rm{C}}{{\rm{E}}_{{\rm{livestock}}}}}}$$

where CEE_farm_, YP_product(*i*)_, PRICE_product(*i*)_, *i*, and *n* represent the carbon economic efficiency (¥/kg CO_2_-eq), yield of products classified as *i* (kg/farm), price of products classified as *i* (¥/kg), product category *i*, and number of product/farm, respectively. CE_crop_ and CE_livestock_ represent the same parameters as in the above equations. All prices of products were based on the mean market price of these products in 2014 and 2015.

### Statistical analyses

The statistical program used in the present research was Genstat19.0 (19th edition; VSN International Ltd, Hemel Hempstead, UK) and SPSS^®^ AMOS 19.0 software’s (IBM Corporation Software Group, Somers, NY, USA). The differences in energy balances, carbon stocks, GHG emissions, carbon economic efficiency, NER, and net income were analyzed using Linear Models, with the four kinds of agricultural production systems fitted as the fixed effect and other parameters as random effects. Predicted means, the standard error of the differences, and the level of significant differences were analyzed using Duncan test. The temporal variations in output indicators among the four systems were also evaluated using a chart presentation. The data of carbon balances and carbon economic efficiency that exhibited high heterogeneity of variance among treatments were transformed in Table 4 to ensure homogeneity of variance using equation log_10_ (data+1) (Xu & He, 2010).

## Results

### Energy balances and net energy ratio of agricultural production

The computed energy balances and NERs are presented in Table 6. For livestock production, input energy and output energy from IFLP were the highest among all four production systems; however, the NER (0.63) for IFLP was the lowest among the three livestock production systems. Of all agriculture production systems in Minqin Oasis, EGLP had the lowest input energy (27.6 GJ/farm). In contrast, the NER (2.74) of the EGLP system was the highest of the four production systems. There were significant differences in energy balances and GHG emissions associated with crop production in Minqin Oasis. The NER of alfalfa (4.01) and maize (2.63) was significantly higher than the corresponding data for other crops (*P* < 0.01) (Table 7).

**Table 6:** Energy balances, net energy ratio, and net income from agricultural production systems in Minqin Oasis.

	ICP	ICLP	IFLP	EGLP	SED^1^	*P*-Value
Energy balances (GJ/Farm)						
Crop						
Input	68.99	54.74	–	–	0.362	<0.001
Output	71.59	70.40	–	–	0.346	<0.001
Balance	2.61	15.66	–	–	0.287	<0.001
NER^2^	1.04	1.29	–	–	0.006	<0.001
Livestock						
Input	–	1.70^c^	201.0^a^	27.6^b^	3.66	<0.001
Output	–	4.3^c^	153.0^a^	75.3^b^	7.77	<0.001
Balance		3.0^b^	−48.5^c^	47.8^a^	2.26	<0.001
NER	–	2.58^a^	0.63^b^	2.74^a^	0.063	<0.001
Crop + Livestock						
Input	72.0^b^	65.0^b^	201.0^a^	27.6^c^	6.24	<0.001
Output	74.0^b^	75.9^b^	153.0^a^	75.3^b^	8.92	<0.001
Balance	2.1^c^	11.7^b^	−48.5^d^	51.8^a^	2.31	<0.001
NER	1.03^c^	1.17^b^	0.63^d^	2.74^a^	0.081	<0.05
Crop and Rangeland (including livestock) (GJ/ha)						
Input	86.58^a^	76.42^b^	–	0.001^c^	1.608	<0.001
Output	89.79^b^	98.97^a^	–	0.002^c^	1.855	<0.001
Balance	3.22^b^	22.55^a^	–	0.001^c^	0.581	<0.001
NER	1.04^c^	1.20^b^	–	2.09^a^	0.02	<0.001
Net income/Farm						
CN¥ (1,000¥)	24.7^d^	32.0^c^	46.4^a^	39.1^b^	9.78	<0.001
US$^3^ (160$)	3.95^d^	5.12^c^	7.42^a^	6.26^b^	1.55	<0.001

**Notes:**

1 SED, standard error of differences.

2 NER, net energy ratio = output energy/input energy.

3 US$: An average of the US$: CN¥ exchange rate for the years 2014–2015 of 1 US$: 6.25 CN¥ has been used to show prices in both currencies (Yahoo! Finance, 2019); similar letters: no significant difference; dissimilar letters (a, b, c, d) indicates a significant difference (*P* < 0.05).

**Table 7:** Energy balances, GHG emissions, carbon economic efficiency, and net energy ratio of crop grown in the Minqin Oasis.

	Wheat (spring)	Maize	Cotton	Sunflower	Chili	Tomato	Melon	Alfalfa	SED^1^	*P*-Value
Energy balances (GJ/ha)										
Input	90.5^c^	76.7^d^	50.2^e^	50.1^e^	101.2^b^	104.9^a^	105.8^a^	44.6^f^	0.38	<0.001
Output	188.5^b^	201.1^a^	70.0^d^	66.0^e^	66.2^e^	66.3^e^	67.0^e^	178.6^c^	1.12	<0.001
Balance	98.3^c^	124.3^b^	19.9^d^	16.2^d^	−34.9^e^	−38.4^f^	−39.4^f^	134.2^a^	1.08	<0.001
NER^2^	2.09^c^	2.63^b^	1.40^d^	1.31^e^	0.66^f^	0.63^f^	0.63^f^	4.01^a^	0.447	<0.001
Carbon balances (ton CO_2_-eq/ha)										
Emissions^3^	10.55^d^	12.79^a^	10.14^e^	12.47^b^	12.24^c^	12.81^a^	12.69^a^	8.73^f^	0.063	<0.001
Stock^4^	12.26^b^	24.59^a^	5.86^e^	7.15^d^	1.44^g^	5.52^f^	0.12^h^	11.64^c^	0.065	<0.001
Balance^5^	1.71^c^	11.81^a^	−4.28^d^	−5.32^e^	−10.80^g^	−7.30^f^	−12.54^h^	2.91^b^	0.149	<0.001
Carbon economic efficiency										
CN¥ (1,000¥/ton CO_2_-eq)	1.79^f^	1.89^e^	2.12^b^	1.55^h^	2.03^d^	3.25^a^	1.77^g^	2.08^c^	0.099	<0.001
US$^6^ (160$/ton CO_2_-eq)	0.29^f^	0.30^e^	0.34^b^	0.25^h^	0.32^d^	0.52^a^	0.28^g^	0.33^c^	0.002	<0.001

**Notes:**

1 SED, standard error of differences.

2 NER, net energy ratio = output energy/input energy.

3 GHG emissions from crop production input.

4 Carbon stock, that is, net deposition of photosynthesis stored by crop products such as grain, stem, and root.

5 Carbon balances of crop production, Balance = stock-emissions.

6 US$: An average of the US$: CN¥ exchange rate for the years 2014–2015 of 1 US$: 6.25 CN¥ has been used to show prices in both currencies (Yahoo! Finance, 2019); similar letters: no significant difference; dissimilar letters (a, b, c, d, e, f, g, h) indicates a significant difference (*P* < 0.05).

### GHG emissions from agricultural production

Greenhouse gas emissions from production input, carbon stocks, and carbon balances of agricultural production systems, per farm (livestock or mixed), and per hectare (farmland), are presented in Table 4. GHG emissions from the EGLP system were significantly higher than those from each of the other three systems (*P* < 0.05), but there were no significant differences between ICP and ICLP. Carbon stock, and carbon balance in the EGLP system were significantly higher than those in each of the other three systems (*P* < 0.05), but there were no significant differences in the other three production systems. At the cropland level, GHG emissions (5.6 ton CO_2_-eq/ha) in EGLP were significantly lower than in ICP and ICLP (*P* < 0.05), but there were no significant differences between ICP and ICLP. The carbon stock (22.2 ton CO_2_-eq/ha) and carbon balance (16.6 ton CO_2_-eq/ha) in the EGLP system were significantly higher than that in ICP and ICLP (*P* < 0.01). The value of carbon stock in ICLP was higher than the corresponding data from the ICP system (*P* < 0.05).

**Figure 3:**
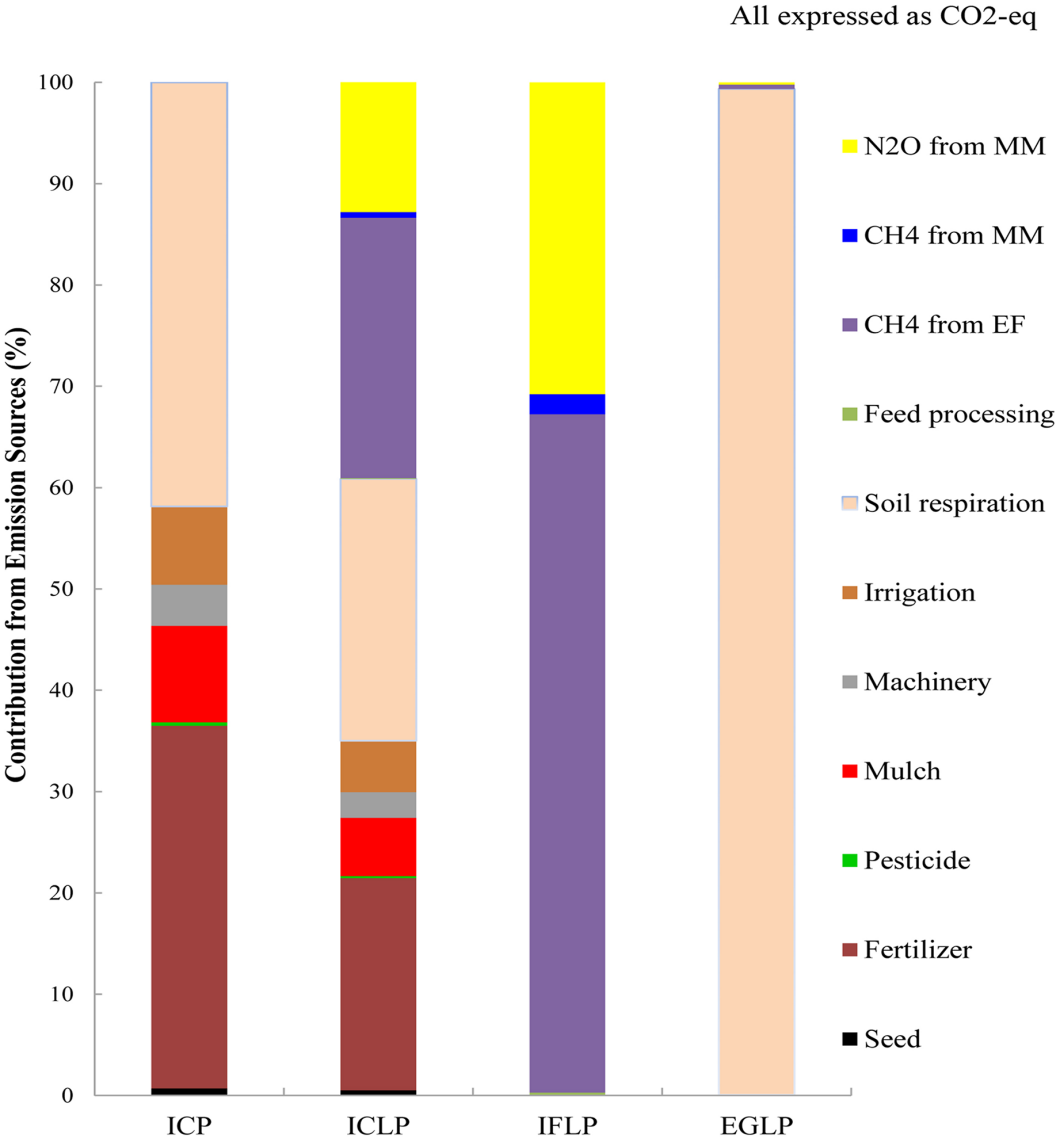
Contribution of all GHG emissions (CO_2_, N_2_O, CH_4_—expressed as CO_2_-eq) from the major farming inputs in Minqin Oasis. MM, manure management; EF, enteric fermentation; ICP, intensive crop production; ICLP, integrated crop-livestock production; IFLP, intensive livestock production (confined feeding); EGLP, extensive livestock production (grazing).

Figure 3 shows the contribution of different factors to the total GHG emission in the abovementioned sub-agricultural systems (namely, ICP, ICLP, IFLP, EGLP) in Minqin Oasis. Among the factors, soil respiration contributes a lot to the total GHG emissions in these sub-systems with the contribution ratio being 41.85% in ICP, 25.86% in ICLP, 99.31% in EGLP, respectively. In the ICP system, fertilizer and mulch resulted in GHG emissions that accounted for 35.78% and 9.53%, respectively. In the ICLP system, methane emissions from enteric fermentation and fertilizer resulted in GHG emissions that accounted for 25.7% and 20.94%, respectively. In the IFLP and EGLP systems, methane emissions and N_2_O emissions accounted for the greater proportion of total GHG emissions; the respective values being as follows:

IFLP ∼ CH_4_: 66.96%; N_2_O: 30.78%; EGLP ∼ CH_4_: 0.42%; N_2_O: 0.21% (Fig. 3).

### Carbon economic efficiency of agricultural production

The carbon economic efficiency of agricultural production in Minqin Oasis is presented in Table 4. That for IFLP was significantly higher than that for each of the other three systems (*P* < 0.05), whereas the differences among the other systems were not significant.

### Net income of agricultural production and analysis of structural equation model

**Figure 4:**
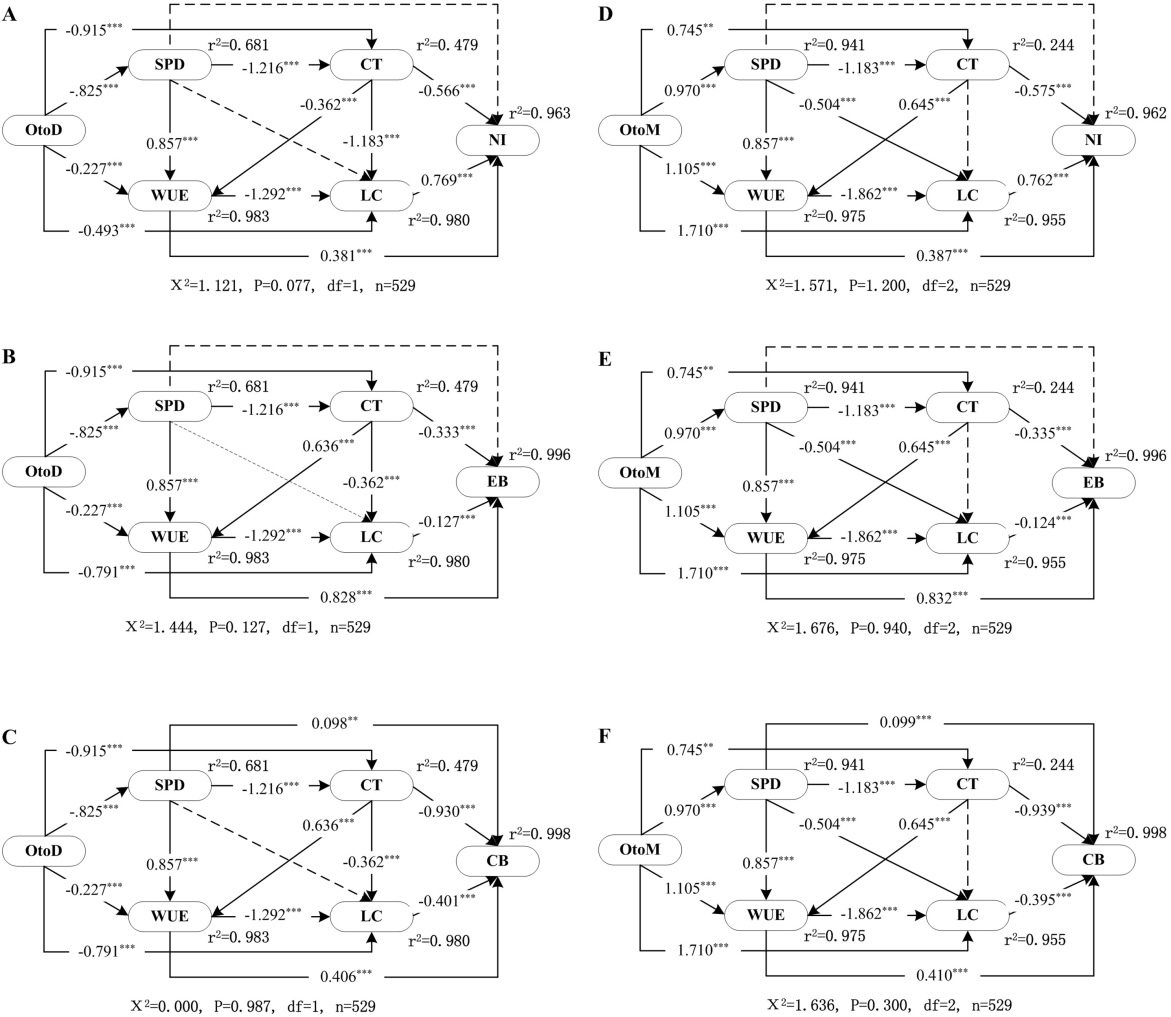
SEM showing the direct and indirect effects of the OtoD on farm (A) net income, (B) energy balance, (C) carbon balances, and the OtoM on farm (D) net income, (E) energy balance, and (F) carbon balances. The models with significant correlation are presented as solid lines. The values on solid lines represent standardized regression weights. Interrupted lines indicate no significant correlation between two variables. Black arrows indicate positive effects. For each endogenous variable the relative amount of explained variance is given. For meanings of abbreviations of variables in oval boxes, see Table 8. χ2, chi-square; *P*, probability level; d*f*, degrees of freedom; *n*, sample size.

The net income of agricultural production in Minqin Oasis is presented in Table 6. Net income for IFLP (1,187.2 US$) was the highest among the four production systems. There were significant differences in net income between other three production systems, as follows ∼ EGLP: 1,001.6 US$; ICLP: 819.2 US$; ICP: 632 US$. The effects between dependent variables and predictor variables were presented in Table 8. The path models showed that the class of livestock was strongly linked to economic income (Fig. 4A, Total effects = 0.769; Fig. 4D, Total effects = 0.762). The direct and total effects of water use efficiency on predicted variables (energy balances, carbon balances) were much stronger than on other dependent variables (Figs. 4B and 4C). Similarly, in path analyses, including the distance from the oasis to mountains as the exogenous variable, the direct and total effects of water use efficiency (through its positive influence on energy and carbon balances), were much stronger than those of other dependent variables (Fig. 4E, Total effects = 1.064; Fig. 4F, Total effects = 1.144).

**Table 8:** The standardized direct, indirect, and total effects between dependent variables and predicted variables.

No. of Fig. 4	Dependent variables	Predicted variables	Direct effects	Indirect effects	Total effects
Figure 4A	OtoD^1^	NI^6^	0.000	0.120	0.120
	SPD^2^	NI	−0.179	0.833	0.654
	CT^3^	NI	−0.566	−0.668	−1.234
	WUE^4^	NI	0.381	−0.994	−0.613
	LC^5^	NI	0.769	0.000	0.769
Figure 4B	OtoD	EB^7^	0.000	−0.904	−0.904
	SPD	EB	0.107	0.456	0.564
	CT	EB	−0.333	0.677	0.343
	WUE	EB	0.828	0.164	0.992
	LC	EB	−0.127	0.000	−0.127
Figure 4C	OtoD	CB^8^	0.000	−0.705	−0.705
	SPD	CB	0.098	1.106	0.924
	CT	CB	−0.93	0.732	−0.198
	WUE	CB	0.406	0.518	1.204
	LC	CB	−0.401	0.000	−0.401
Figure 4D	OtoM^9^	NI	0.000	0.102	0.102
	SPD	NI	−0.182	0.885	0.703
	CT	NI	−0.575	−0.498	−1.073
	WUE	NI	0.387	−1.419	−1.031
	LC	NI	0.762	0.000	0.762
Figure 4E	OtoM	EB	0.000	0.941	0.941
	SPD	EB	0.108	−0.32	−0.212
	CT	EB	−0.335	0.659	0.323
	WUE	EB	0.832	0.232	1.064
	LC	EB	−0.124	0.000	−0.124
Figure 4F	OtoM	CB	0.000	0.933	0.933
	SPD	CB	0.099	0.54	0.639
	CT	CB	−0.939	0.651	−0.288
	WUE	CB	0.41	0.734	1.144
	LC	CB	−0.395	0.000	−0.395

**Notes:**

Gray highlight indicates the greatest positive direct effect, indirect effect, and total effect between dependent and independent variables.

1 OtoD, the distance from oasis to desert (km).

2 SPD, soil particle diameter (μm).

3 CT, crop type.

4 WUE, water use efficiency (MJ/m^3^).

5 LC, livestock category.

6 NI, net income (1,000¥/farm).

7 EB, energy balances (GJ/farm).

8 CB, carbon balance, that is, carbon stock—GHG emissions from production input (ton CO_2_-eq/farm).

9 OtoM, the distance from oasis to mountain (km).

## Discussion

### Energy balances and net energy ratio of agricultural production systems

The energy balance of agricultural production systems can be influenced by variations in farm input and output capacities, including family population, production systems, environmental conditions, management regimes, and input capacity. It is known that the evaluation of energy balances is related to the variability of computed energy parameters. The parameters of energy and GHG emissions in this study were collected from literature in similar research sites. For example, the energy parameters of herbicides and insecticides selected in this study are higher than that reported by Pishgar-Komleh, Ghahderijani & Sefeedpari (2012b). The present carbon balances for agricultural production are comparable to those published elsewhere. For example, our NER for wheat and maize production are similar to those in Iran (2.09 vs. 2.13 GJ/ha, 2.63 vs. 2.67 GJ/ha, respectively) (Khoshroo, 2014; Yousefi, Damghani & Khoramivafa, 2014). However, our input energy and output energy of maize production (76.7 and 201.1 GJ/ha, respectively) are much higher than those (50.5 and 134.9 GJ/ha, respectively) estimated using LCA in Iran (Yousefi, Damghani & Khoramivafa, 2014). Our input energy for cotton production (50.2 GJ/ha) is much higher than that (31.2 GJ/ha) in Iran (Pishgar-Komleh, Sefeedpari & Ghahderijani, 2012a). The present NER of ICP (1.04) and IFLP (0.63) are within the range of crop production (0.5–2.1 GJ/farm) and livestock production (0.5–1.0 GJ/farm) of eastern Gansu in China, respectively (Xu et al., 2010). Our NER for tomato production (0.63) is similar to that (0.6) in India (Nautiyal et al., 2007). However, the NER of wheat production (2.09) in this study is higher than that in Pakistan (Abbas et al., 2017), and the corresponding value of maize and cotton production in our study are much lower than that (2.63 vs. 5.52) in Turkey and (1.40 vs. 2.27) in India, respectively (Baran & Gokdogan, 2016; Channagouda, Babalad & Patil, 2017).

The nature of agricultural production systems is the flow and circulation of matter and energy (Sere, Steinfeld & Groenewold, 1996). Energy is the foundation of the development of agricultural systems. ICP, which is an open system in Minqin Oasis, depends on high inputs with fertilizer, plastic mulch, and machinery accounting for 99% the total inputs. The large input of inorganic energy has improved the living standards of local farmers, however, the inorganic energy, especially, chemical fertilizer, pesticide and plastic mulch have yielded negative effect on local environment. It is a sustainable mode of agricultural development to enlarge the alfalfa planting area and to breed numerous sheep in Minqin Oasis.

### Carbon balances of agricultural production systems

As indicated previously, our GHG emission factors are comparable to those published elsewhere. For example, the average value of the carbon balance for grassland from intensive livestock production (Grazing) in Minqin Oasis is higher than that (49.1 vs. 22–44 g/C/m^2^.year) for grassland in southern Belgium (Goidts & Van Wesemael, 2007), and lower than that (129 g C/m^2^.year) for grazed European grassland. Our carbon emission for maize production (12.79 ton CO_2_-eq/ha) is similar to that (12.865 ton CO_2_-eq/ha) reported in Iran (Soussana, Tallec & Blanfort, 2010). Similar findings were reported, that is, that the restoration and reconstruction of grassland can significantly increase the amount of soil organic carbon storage in China (Li et al., 2006). The present carbon economic efficiency ($0.0464/kg CO_2_-eq) is within the range for wheat production ($0.01–$0.085/kg CO_2_-eq) in the USA (Sanders & Webber, 2014). Nevertheless, our GHG emission factor for wheat production is higher than that (10.55 vs. 1.28 ton CO_2_-eq/ha) in the UK (Hillier et al., 2009); for maize production is higher that (12.79 vs. 2.44–4.20 ton CO_2_-eq/ha) in the USA (Farrell et al., 2006).

There is no similar research on energy and carbon balances, which are of great significance to adjust the agricultural production model in China. The high inputs, such as fertilizer, mulch, and machining, accounted for a relatively large proportion, and low outputs in crop production resulted in high carbon emission in Minqin Oasis. It was found that the agricultural production inputs, that is, fertilizer and plastic mulch are the dominant factors that contribute to GHG emissions in this study. In Hexi corridor (including Minqin Oasis) of China, the average annual growth rate of agricultural fertilizer and film per unit of area had a slight increase since 2012 and reached to a certain extent (Li et al., 2016). Therefore, this finding can well provide suggestions to policy makers to adjust agricultural production model in Minqin Oasis, China. In addition, GHG emissions might be assigned a price in prospective climate policy frameworks. It would be useful to know the extent to which those policies would increase the incremental production costs of crop production within the agricultural production system.

### Uncertainty of GHG emissions assessment

Many factors could contribute to the uncertainty of the present assessment of GHG emissions from typical agricultural production systems in Minqin Oasis. First, although the eight towns selected from each production system were typical of the production system in the region, these eight towns might not fully cover all variations in crop and livestock production systems within each region. Second, the official data collection system in China might not be as good as that in developed countries (Xue, Wang & Yan, 2014). In addition, the emission factors of the seed, P and K fertilizers, and pesticides in China were estimated using reported values (Cheng et al., 2011) and (Zeng et al., 2012), which originated from other countries. The use of the Tier 1 method proposed by the Intergovernmental Panel on Climate Change (IPCC) 2013 (Intergovernmental Panel on Climate Change (IPCC), 2014) also added uncertainty to the present emission factors for livestock production because this method does not consider the effects of animals and dietary factors on enteric methane emissions. In summary, although the above uncertainties might add errors to estimates of GHG emissions in Minqin Oasis, our results could provide benchmark information for the Chinese government to develop appropriate policies to reduce GHG emissions from agricultural production in northwestern China. However, further improvement is required in future to upgrade the current evaluation of GHG emissions from agricultural production systems in this area.

## Conclusions

The present study developed models to estimate energy balances and GHG emissions within the farm gate associated with the production per farm for the four contrasting agricultural production systems in Minqin Oasis. The statistical analysis of data from 2014 to 2015 indicated that the NER in EGLP was significantly higher than that in other three systems. The current research found that the EGLP system in Minqin Oasis is a carbon sink, and the net income in IFLP was the highest among the four systems in Minqin Oasis. However, relative to the contribution of GHG emissions from production input, all of the results of the four agricultural systems showed that fertilizer, methane emissions from enteric fermentation, and plastic mulch accounted for the greatest proportion. The path models showed that breeding structure was strongly linked to the economic income. The direct and total effects of water use efficiency via its positive influences on energy balances and GHG emissions were much stronger than those of other dependent variables. Although there is a range of uncertainties relating to the calculations of these emission factors, these data could provide benchmark information for Chinese authorities to evaluate the effect of GHG emissions from contrasting agricultural production systems in Minqin Oasis.

## Supplemental Information

10.7717/peerj.6890/supp-1 Supplemental Information 1 Raw data exported from the farm survey applied for Tables 1, 4, 6, 7 and Figure 3.

10.7717/peerj.6890/supp-2 Supplemental Information 2 The structured questionnaire of farm survey.

10.7717/peerj.6890/supp-3 Supplemental Information 3 SEM information collected from public literature and farmer interview.

10.7717/peerj.6890/supp-4 Supplemental Information 4 GHG emissions, carbon stock, carbon balance, and carbon economic efficiency of agricultural production systems in Minqin Oasis. ^1^SED: standard error of differences; ^2^GHG emissions from crop production inputs; ^3^carbon stock of the net accumulation of photosynthesis from crop products, such as the grain, stem, and root; ^4^carbon balances of crop production (carbon stock–GHG emissions); ^5^GHG emissions from livestock production; ^6^carbon stock from livestock products, such as the carcass, milk and wool; ^7^carbon balances of livestock production (carbon stock–GHG emissions); ^8^US$: An average of the US$: CN¥ exchange rate for the years 2014 to 2015 of 1 US$: 6.25 CN¥ has been used to show prices in both currencies (http://finance.yahoo.com/chart/USDCNY); similar letters: no significant difference; dissimilar letters (a, b, c) indicates a significant difference (*P* < 0.05).

## Additional Information and Declarations

### Data Availability

The following information was supplied regarding data availability: Raw data are available in File S1.

## References

[ref-1] Abbas A, Yang ML, Ahmad R, Yousaf K, Iqbal T (2017). Energy use efficiency in wheat production, a case study of Punjab Pakistan. Fresenius Environmental Bulletin.

[ref-2] Adom F, Maes A, Workman C, Clayton-Nierderman Z, Thoma G, Shonnard D (2012). Regional carbon footprint analysis of dairy feeds for milk production in the USA. International Journal of Life Cycle Assessment.

[ref-3] Arbuckle J (2010). IBM SPSS Amos 19 user’s guide.

[ref-4] Baran MF, Gokdogan O (2016). Comparison of energy use efficiency of different tillage methods on the secondary crop corn silage production. Fresenius Environmental Bulletin.

[ref-5] Blook H, Kool A, Luske B, Scholten TP (2010). Methodology for assessing carbon footprints of horticultural products.

[ref-6] Castillo EF, Mora M (2000). Mathematical modelling as a tool for environmental evaluation of industrial sectors in Colombia. Waste Management.

[ref-7] Channagouda RF, Babalad HB, Patil RK (2017). Impact of organic manures, green leaf manures and micronutrients on natural enemies and energy use efficiency in cotton. Indian Journal of Agricultural Sciences.

[ref-8] Chen F, Gai AH, Li CB (2009). Soil organic carbon storage and its spatial distribution in Gansu Province. Journal of Arid Land Resources & Environment.

[ref-9] Chen S, Huang Y, Zou J, Shen Q, Hu Z, Qin Y, Chen H, Pan G (2010). Modeling interannual variability of global soil respiration from climate and soil properties. Agricultural and Forest Meteorology.

[ref-10] Cheng K, Pan G, Smith P, Luo T, Li L, Zheng J, Zhang X, Han X, Yan M (2011). Carbon footprint of China’s crop production—an estimation using agro-statistics data over 1993–2007. Agriculture, Ecosystems & Environment.

[ref-11] Dazhong W, Pimentel D (1984). Energy flow through an organic agroecosystem in China. Agriculture Ecosystems & Environment.

[ref-12] Dong HM, Li YE, Tao XP, Peng XP, Li N, Zhu ZP (2008). China greenhouse gas emissions from agricultural activities and its mitigation strategy. Transactions of the Chinese Society of Agricultural Engineering.

[ref-13] Dubey A, Lal R (2009). Carbon footprint and sustainability of agricultural production systems in Punjab, India, and Ohio, USA. Journal of Crop Improvement.

[ref-14] Dyer JA, Desjardins RL (2006). Carbon dioxide emissions associated with the manufacturing of tractors and farm machinery in Canada. Biosystems Engineering.

[ref-15] Farrell AE, Plevin RJ, Turner BT, Jones AD, O’Hare M, Kammen DM (2006). Ethanol can contribute to energy and environmental goals. Science.

[ref-16] Ghorbani R, Mondani F, Amirmoradi S, Feizi H, Khorramdel S, Teimouri M, Sanjani S, Anvarkhah S, Aghel H (2011). A case study of energy use and economical analysis of irrigated and dryland wheat production systems. Applied Energy.

[ref-17] Goidts E, Van Wesemael B (2007). Regional assessment of soil organic carbon changes under agriculture in Southern Belgium (1955–2005). Geoderma.

[ref-18] Gollnow S, Lundie S, Moore AD, McLaren J, Van Buuren N, Stahle P, Christie K, Thylmann D, Rehl T (2014). Carbon footprint of milk production from dairy cows in Australia. International Dairy Journal.

[ref-19] Grace JB (2006). Structural equation modeling and natural systems.

[ref-20] He FY, Wang JH, Gao ZH, Xu YS, Ma QL (2004). Grassland agriculture research in the Hexi desert oasis region—a case study of Minqin Oasis. Acta Pratacultural Science.

[ref-21] Hillier J, Hawes C, Squire GR, Hilton AJ, Wale SJ, Smith P (2009). The carbon footprints of food crop production. International Journal of Agricultural Sustainability.

[ref-22] Hou FJ, Chang SH, Nan ZB (2009). Establish the pastoral agriculture system for desertification control in Minqin. Pratacultural Science.

[ref-23] Hou FJ, Nan ZB, Xie YZ, Li XL, Lin HL, Ren JZ (2008). Integrated crop-livestock production systems in China. Rangeland Journal.

[ref-24] Huang J, Ji M, Xie Y, Wang S, He Y, Ran J (2016). Global semi-arid climate change over last 60 years. Climate Dynamics.

[ref-25] Huang ZW, Yang DG, Li XP (2004). Analysis on the energy flow of the farmer households and the characteristics of the eco-economic fractals in the middle and lower reaches of the Tarim river. Arid Zone Research.

[ref-26] Intergovernmental Panel on Climate Change (IPCC) (2006). IPCC guidelines for National Greenhouse Gas Inventories. https://www.ipcc-nggip.iges.or.jp/public/2006gl/index.html.

[ref-27] Intergovernmental Panel on Climate Change (IPCC) (2014). Climate change 2014: mitigation of climate change. http://www.ipcc.ch/report/ar5/wg3.

[ref-29] International Organization for Standardization (ISO). ISO14044 (2006). Environmental management—life cycle assessment—requirements and guidelines.

[ref-28] Iriarte A, Villalobos P (2013). Greenhouse gas emissions and energy balance of sunflower biodiesel: identification of its key factors in the supply chain. Resources, Conservation and Recycling.

[ref-30] Joint Research Centre of the European Commission (JRC) (2010). ILCD handbook: general guide for life cycle assessment—detailed guidance.

[ref-31] Khoshnevisan B, Rafiee S, Omid M, Mousazadeh H, Rajaeifar MA (2014). Application of artificial neural networks for prediction of output energy and GHG emissions in potato production in Iran. Agricultural Systems.

[ref-32] Khoshroo A (2014). Energy use pattern and greenhouse gas emission of wheat production: a case study in Iran. Agricultural Communications.

[ref-33] Lal R (2004). Carbon emission from farm operations. Environment International.

[ref-34] Lal R (2010). Enhancing crop yields in the developing countries through restoration of the soil organic carbon pool in agricultural lands. Land Degradation & Development.

[ref-35] Li SX, Liu JY, Zhang L, Cheng JH (2013). Coal consumption, carbon emission and regional economic performance across 13 major provinces. Resources Science.

[ref-36] Li XL, Zhang XT, Niu J, Tong L, Kang SZ, Du TS, Li S, Ding RS (2016). Irrigation water productivity is more influenced by agronomic practice factors than by climatic factors in Hexi Corridor, Northwest China. Scientific Reports.

[ref-37] Li YQ, Zhao HL, Zhao XY, Zhang TH, Chen YP (2006). Soil respiration, carbon balance and carbon storage of sandy grassland under post-grazing natural restoration. Acta Prataculturae Sinica.

[ref-38] Liu HQ, Fu JX, Liu SY, Xie XY, Yang XY (2016). Calculation methods and application of carbon dioxide emission during steel-making process. Iron & Steel.

[ref-39] Liu YJ, Li X, Tian GF, Wu ZX (2017). SIEMENS expert optimization control system for cement production line. Cement Engineering.

[ref-40] Lu FB (1994). Energy flow in the agroecosystem of farming-livestock-fruit. Eco-Agriculture Research.

[ref-41] Lu F, Wang XK, Han B, Ouyang ZY, Duan XN, Zheng H (2008). Assessment on the availability of nitrogen fertilization in improving carbon sequestration potential of China’s cropland soil. Chinese Journal of Applied Ecology.

[ref-42] Meng XH, Cheng GQ, Zhang JB, Wang Y, Zhou HC (2014). Analyze on the spatial temporal characteristics of GHG estimation of livestock’s by life cycle assessment in China. China Environmental Science.

[ref-43] Miao GY, Yin J, Zhang YT, Zhang AL (1998). Study on root growth of main crops in North China. Acta Agronomica Sinica.

[ref-44] Nautiyal S, Kaechele H, Rao KS, Maikhuri RK, Saxena KG (2007). Energy and economic analysis of traditional versus introduced crops cultivation in the mountains of the Indian Himalayas: a case study. Energy.

[ref-45] Nautiyal S, Maikhuri RK, Semwal RL, Rao KS, Saxena KG (1998). *Agroforestry Systems* in the rural landscape—a case study in Garhwal Himalaya, India. India Agroforestry Systems.

[ref-46] Ni J (2001). Carbon storage in terrestrial ecosystems of China: estimates at different spatial resolutions and their responses to climate change. Climatic Change.

[ref-47] Ozkan B, Akcaoz H, Fert C (2004). Energy input-output analysis in Turkish agriculture. Renewable Energy.

[ref-48] Pimentel D (1980). Handbook of energy utilization in agriculture.

[ref-49] Piñero P, Cazcarro I, Arto I, Mäenpää I, Juutinen A, Pongrácz E (2018). Accounting for raw material embodied in imports by multi-regional input-output modelling and life cycle assessment, using Finland as a study case. Ecological Economics.

[ref-50] Pishgar-Komleh SH, Ghahderijani M, Sefeedpari P (2012b). Energy consumption and CO_2_ emissions analysis of potato production based on different farm size levels in Iran. Journal of Cleaner Production.

[ref-51] Pishgar-Komleh SH, Sefeedpari P, Ghahderijani M (2012a). Exploring energy consumption and CO_2_ emission of cotton production in Iran. Journal of Renewable and Sustainable Energy.

[ref-52] Qi Y, Huang Y, Wang Y, Zhao J, Zhang J (2011). Biomass and its allocation of four grassland species under different nitrogen levels. Acta Ecologica Sinica.

[ref-53] Reichmann LG, Sala OE (2015). Differential sensitivities of grassland structural components to changes in precipitation mediate productivity response in a desert ecosystem. Functional Ecology.

[ref-54] Ren JZ, Lin HL, Wei L (2009). Grassland farming is an important approach for the sustainable development of agriculture in Gansu province. Acta Agrestia Sinica.

[ref-55] Ren J, Wan C (1994). System coupling and desert-oasis agro-ecosystem. Acta Pratacultural Science.

[ref-56] Sanders KT, Webber ME (2014). A comparative analysis of the greenhouse gas emissions intensity of wheat and beef in the United States. Environmental Research Letters.

[ref-57] Sere C, Steinfeld H, Groenewold J (1996). World livestock production systems: food and agricultural organization of the united nations (FAOUN). http://www.fao.org/3/a-w0027e.pdf.

[ref-58] Shi LG, Chen F, Kong FL (2011a). The carbon footprint of winter wheat-summer maize cropping pattern on north China plain. China Population Resources & Environment.

[ref-59] Shi LG, Fan SC, Kong FL, Chen F (2011b). Preliminary study on the carbon efficiency of main crops production in north China plain. Acta Agronomica Sinica.

[ref-60] Soussana JF, Tallec T, Blanfort V (2010). Mitigating the greenhouse gas balance of ruminant production systems through carbon sequestration in grasslands. Animal.

[ref-61] Tian Y, Zhang JB (2013). Regional differentiation research on net carbon effect of agricultural production in China. Journal of Natural Resources.

[ref-62] Wang W, Koslowski F, Nayak DR, Smith P, Saetnan E, Ju X, Guo L, Han G, De Perthuis C, Lin E (2014). Greenhouse gas mitigation in Chinese agriculture: distinguishing technical and economic potentials. Global Environmental Change.

[ref-63] Wang PC, Liu Q, Wang Y, Qiong MA (2017). The assessment of agricultural waste resources recycling way based on ecological footprint theory: a case study of cotton straw in Southern Xinjiang. Ecological Economy.

[ref-64] Wang JQ, Lu DX, Yang HJ, Yang ZB, Luo QJ, Yang YF, Wang HR, Xiong BH, Zhang L, Qu XX, Zhen ZC, Mao YY (2004). Agricultural standards—feeding standard of meat producting sheep and goats (NY/T 816-2004). Hunan Forage.

[ref-65] West TO, Marland G (2002). A synthesis of carbon sequestration, carbon emissions, and net carbon flux in agriculture: comparing tillage practices in the United States. Agriculture, Ecosystems & Environment.

[ref-66] Wu CC, Gao XY, Hou FJ (2017). Carbon balance of household production system in the transition zone from the Loess Plateau to the Qinghai-Tibet Plateau, China. Chinese Journal of Applied Ecology.

[ref-67] Xu XH, He MZ (2010). Experimental design, design-expert & SPSS.

[ref-68] Xu L, Wang XY, Hou FJ, Nan ZB (2010). Energy balance of intergraded crop/rangeland-livestock production systems in eastern Gansu, China. http://www.regional.org.au/au/asa/2010/farming-systems/international/7071_xul.htm.

[ref-69] Xue B, Wang LZ, Yan T (2014). Methane emission inventories for enteric fermentation and manure management of yak, buffalo and dairy and beef cattle in China from 1988 to 2009. Agriculture, Ecosystems & Environment.

[ref-73] Yahoo! Finance (2019). Symbols similar to ‘usdcny’. https://finance.yahoo.com/lookup?s=USDCNY.

[ref-70] Yousefi M, Damghani AM, Khoramivafa M (2014). Energy consumption, greenhouse gas emissions and assessment of sustainability index in corn agroecosystems of Iran. Science of the Total Environment.

[ref-71] Yuan S, Peng SB, Wang D, Man JG (2018). Evaluation of the energy budget and energy use efficiency in wheat production under various crop management practices in China. Energy.

[ref-72] Zeng XF, Zhao SW, Li XX, Li T, Liu J (2012). Main crops carbon footprint in Pingluo county of the Ningxia hui autonomous region. Bulletin of Soil & Water Conservation.

