# Supplementary material for: Evaluation of energy balances and greenhouse gas emissions from different agricultural production systems in Minqin Oasis, China
Source: PeerJ. 2019 Jun 26;7:e6890. doi: 10.7717/peerj.6890 (PMC6599452; doi:10.7717/peerj.6890)
Supplement: Supplemental Information 2 [file peerj-07-6890-s002.docx]

| **Table S1 The structured questionnaire of farm survey.** | | |
| --- | --- | --- |
| **Data** | **Units** | **Source** |
| **Crop production** | | |
| labor type | - | farmer interview |
| labor input | hours/year | farmer interview |
| crop type | (e. g. maize) | farmer interview |
| sowing area | ha | farmer interview |
| seed source | - | farmer interview |
| amount of seeds used | kg/ha | farmer interview |
| rate of fertilizers used | kg/ha | farmer interview |
| rate of pesticide used | kg/ha | farmer interview |
| fuel consumption for production | kg/ha | farmer interview |
| amount of plastic film | kg/ha | farmer interview |
| farm machine type | - | farmer interview |
| farm machine (life and working hours) | hours/year | farmer interview |
| electricity consumption for irrigation | kWh/year | farmer interview |
| yield of crop product | kg/ha | farmer interview |
| yield of crop straw | kg/ha | farmer interview |
| market price of inputs | ¥/kg | farmer interview, local government officials |
| market price of outputs | ¥/kg | farmer interview, local government officials |
| net income | 1,000¥/farm | farmer interview |
| **Livestock production** | | |
| labor type | - | farmer interview |
| labor input | hours/year | farmer interview |
| category | (e.g. sheep) | farmer interview |
| numbers classified livestock | head/farm | farmer interview |
| age classified livestock | month/head | farmer interview |
| weight classified livestock | kg/head | farmer interview |
| carcass weight classified livestock | kg/head | farmer interview |
| milk | kg/head | farmer interview |
| wool | kg/head | farmer interview |
| feed source | - | farmer interview |
| feed type | (e.g. hay, forage) | farmer interview |
| feed usage | kg/head | farmer interview |
| veterinary drug usage | kg/head | farmer interview |
| lighting of housing structures | kWh/farm | farmer interview |
| coal consumption of housing structures | kg/farm | farmer interview |
| market price of inputs | ¥/kg | farmer interview, local government officials |
| market price of outputs | ¥/kg | farmer interview, local government officials |
| net income | 1,000¥/farm | farmer interview |
