# Supplementary material for: Evaluation of energy balances and greenhouse gas emissions from different agricultural production systems in Minqin Oasis, China
Source: PeerJ. 2019 Jun 26;7:e6890. doi: 10.7717/peerj.6890 (PMC6599452; doi:10.7717/peerj.6890)
Supplement: Supplemental Information 3 [file peerj-07-6890-s003.docx]

| **Table S2 SEM information collected from public literature and farmer interview.** | | | |
| --- | --- | --- | --- |
| **Node** | **Node Name** | **Units** | **Source** |
| OtoD | distance from oasis to desert | km. | farmer interview |
| OtoM | distance from oasis to mountain | km. | farmer interview |
| SPD | soil particle diameter | μm | public literature |
| CT | crop type | (e.g. maize) | farmer interview |
| CL | class of livestock | (e.g. sheep) | farmer interview |
| WUE | water use efficiency | MJ/m^3^ | public literature |
| NI | net income | 1,000¥/farm | farmer interview |
| EB | energy balances | GJ/farm | calculated |
| CB | carbon balances | kg CO_2_-eq/farm | calculated |
