# Supplementary material for: Evaluation of energy balances and greenhouse gas emissions from different agricultural production systems in Minqin Oasis, China
Source: PeerJ. 2019 Jun 26;7:e6890. doi: 10.7717/peerj.6890 (PMC6599452; doi:10.7717/peerj.6890)
Supplement: Supplemental Information 4 — 1SED: standard error of differences; 2GHG emissions from crop production inputs; 3carbon stock of the net accumulation of photosynthesis from crop products, such as the grain, stem, and root; 4carbon balances of crop production (carbon stock–GHG emissions); 5GHG emissions from livestock production; 6carbon stock from livestock products, such as the carcass, milk and wool; 7carbon balances of livestock production (carbon stock–GHG emissions); 8US$: An average of the US$: CN¥ exchange rate for the years 2014 to 2015 of 1 US$: 6.25 CN¥ has been used to show prices in both currencies (http://finance.yahoo.com/chart/USDCNY); similar letters: no significant difference; dissimilar letters (a, b, c) indicates a significant difference (P < 0.05). [file peerj-07-6890-s004.docx]

| **Table S3 GHG emissions, carbon stock, carbon balance, and carbon economic efficiency of agricultural production systems in Minqin Oasis.** | | | | | | |
| --- | --- | --- | --- | --- | --- | --- |
|  | ICP | ICLP | IFLP | EGLP | SED^1^ | P-Value |
| Carbon balance (tonne CO_2_-eq /farm) | | | | | | |
| Crop & Rangeland (not including livestock) | | | | | | |
| GHG emissions^2^ | 10.2^b^ | 9.2^b^ | - | 9,978.0^a^ | 31.02 | <0.001 |
| Carbon stock^3^ | 7.6^b^ | 8.0^b^ | - | 39,430.0^a^ | 125.40 | <0.001 |
| Carbon balance^4^ | -2.5^b^ | -0.7^b^ | - | 29,451.0^a^ | 96.30 | <0.001 |
| Livestock | | | | | | |
| GHG emissions^5^ | - | 7.3^c^ | 191.8^a^ | 79.1^b^ | 1.59 | <0.001 |
| Carbon stock^6^ | - | 0.25^c^ | 6.48^a^ | 2.68^b^ | 0.049 | <0.001 |
| Carbon balance^7^ | - | -7.07^a^ | -185.33^c^ | -76.45^b^ | 1.553 | <0.001 |
| Crop & Rangeland (including livestock) | | | | | | |
| GHG emissions | 10.2^c^ | 16.0^c^ | 192.0^b^ | 10,058.0^a^ | 27.13 | <0.001 |
| Carbon stock | 7.6^b^ | 9.0^b^ | 6.0^b^ | 39,432.0^a^ | 109.51 | <0.001 |
| Carbon balance | -2.5^b^ | -8.0^b^ | -185.0^c^ | 29,375.0^a^ | 84.00 | <0.001 |
| Carbon economic efficiency (1,000 CN¥/tonne CO_2_-eq /farm) | | | | | | |
| CN¥ (1000¥/tonne CO_2_-eq /farm) | | | | | | |
| Crop & Rangeland  (not including livestock) | 5.12^a^ | 5.24^a^ | - | 3.26^b^ | 0.041 | <0.001 |
| Livestock | - | 2969.0^a^ | 2771.0^b^ | 3,015.0^a^ | 25.94 | <0.001 |
| Crop & Rangeland (including livestock) | 5.12^b^ | 10.19^b^ | 2771.00^a^ | 93.80^b^ | 40.401 | <0.001 |
| US$^8^(160$/tonne CO_2_-eq /farm) | | | | | | |
| Crop & Rangeland  (not including livestock) | 0.82^a^ | 0.84^a^ | - | 0.52^b^ | 0.006 | <0.001 |
| Livestock | - | 475.1^a^ | 443.3^b^ | 482.4^a^ | 4.15 | <0.001 |
| Crop & Rangeland (including livestock) | 0.82^b^ | 1.63^b^ | 443.30^a^ | 8.81^b^ | 6.46 | <0.001 |
| Crop & Rangeland (including livestock) (tonne CO_2_-eq /ha) | | | | | | |
| GHG emissions | 12.7^a^ | 12.6^a^ | - | 5.6^b^ | 0.04 | <0.001 |
| Carbon stock | 9.6^c^ | 12.1^b^ | - | 22.2^a^ | 0.08 | <0.001 |
| Carbon balance | -3.2^c^ | -0.6^b^ | - | 16.6^a^ | 0.07 | <0.001 |
| ^1^SED: standard error of differences; ^2^GHG emissions from crop production inputs; ^3^carbon stock of the net accumulation of photosynthesis from crop products, such as the grain, stem, and root; ^4^carbon balances of crop production (carbon stock - GHG emissions); ^5^GHG emissions from livestock production; ^6^carbon stock from livestock products, such as the carcass, milk and wool; ^7^carbon balances of livestock production (carbon stock - GHG emissions); ^8^US$: An average of the US$ : CN¥ exchange rate for the years 2014 to 2015 of 1 US$ : 6.25 CN¥ has been used to show prices in both currencies (http://finance.yahoo.com/chart/USDCNY); similar letters: no significant difference; dissimilar letters (a, b, c) indicates a significant difference (*P*<0.05). | | | | | | |
